# Phase-enabled metal-organic framework homojunction for highly selective CO_2_ photoreduction

**DOI:** 10.1038/s41467-021-21401-2

**Published:** 2021-02-23

**Authors:** Yannan Liu, Chuanshuang Chen, Jesus Valdez, Debora Motta Meira, Wanting He, Yong Wang, Catalin Harnagea, Qiongqiong Lu, Tugrul Guner, Hao Wang, Cheng-Hao Liu, Qingzhe Zhang, Shengyun Huang, Aycan Yurtsever, Mohamed Chaker, Dongling Ma

**Affiliations:** 1 Énergie Matériaux et Télécommunications, Institut National de la Recherche Scientifque (INRS), Varennes, QC Canada; 2 School of Chemistry and Chemical Engineering, Frontiers Science Center for Transformative Molecules, Shanghai Jiao Tong University, Shanghai, PR China; 3 CLS@APS sector 20, Advanced Photon Source, Argonne National Laboratory, Lemont, IL USA; 4 Canadian Light Source Inc., Saskatoon, SK Canada; 5 Leibniz Institute for Solid State and Materials Research (IFW) Dresden e.V., Dresden, Germany; 6 Department of Chemistry, McGill University, Montreal, QC Canada

**Keywords:** Metal-organic frameworks, Photocatalysis, Composites, Nanoscale materials

## Abstract

Conversion of clean solar energy to chemical fuels is one of the promising and up-and-coming applications of metal–organic frameworks. However, fast recombination of photogenerated charge carriers in these frameworks remains the most significant limitation for their photocatalytic application. Although the construction of homojunctions is a promising solution, it remains very challenging to synthesize them. Herein, we report a well-defined hierarchical homojunction based on metal–organic frameworks via a facile one-pot synthesis route directed by hollow transition metal nanoparticles. The homojunction is enabled by two concentric stacked nanoplates with slightly different crystal phases. The enhanced charge separation in the homojunction was visualized by in-situ surface photovoltage microscopy. Moreover, the as-prepared nanostacks displayed a visible-light-driven carbon dioxide reduction with very high carbon monooxide selectivity, and excellent stability. Our work provides a powerful platform to synthesize capable metal–organic framework complexes and sheds light on the hierarchical structure-function relationships of metal–organic frameworks.

## Abstract (web summary)

Homojunctions are very promising in photocatalysis, but challenging to achieve. Herein, authors report a well-defined hierarchical metal–organic framework-based homojunction, formed via a one-pot synthesis route directed by hollow transition metal nanoparticles, as photocatalysts for CO_2_ reduction.

## Introduction

Metal–organic frameworks (MOFs), a class of emerging crystalline, coordination polymers, are built from metal ions or clusters linked with organic ligands^1–3^. In virtue of the tailorable chemistry, uniform porosity, and ordered crystalline structures^4–7^, MOFs have shown great potential in catalysis^8–10^. Unlike traditional bulk MOFs with a broad size distribution spanning several orders of magnitude (from millimeter to nanometer), reducing the size of MOFs to nanoscale in one or more dimensions exhibits advantages such as accelerated adsorption–desorption kinetics and promoted mass transfer in catalysis. It also allows for the construction of well-defined hierarchical nanostructures^11–13^, which has significance for in-depth understanding of MOFs’ structure-function relationships and achieving high catalytic activity. However, it is still challenging to build MOFs with precisely controllable complex structures in nanoscale.

Photocatalytic conversion of CO_2_ to chemical fuels using sustainable and abundant solar energy is attractive for relieving both global warming and energy issues^14–17^. Much effort has been devoted to developing MOFs-based semiconductors for photocatalysis^18–20^. However, the severe recombination of photogenerated carriers and poor selectivity toward reaction products limited their practical application^21,22^. Homojunctions, composed of semiconductors with analogous composition but different energy levels^23,24^, have attracted more and more attention for their superior capability of promoting charge separation as compared to traditional heterojunctions in recent years, due to their features of faster carrier transfer through chemically similar interfaces^25–27^. Although some organic- and inorganic-based homojunctions have been well developed, to the best of our knowledge, promising versatile MOF-based hierarchical structures have not yet been successfully exploited in homojunctions. Besides, most of the reported homojunctions involve elaborate multiple-step manipulation, such as copper-surface-mediated laser coupling method^25^, combined deposition processing approach^26^ and programmed exfoliation procedure^27^. Thus, it is of considerable significance to develop MOF-based homojunctions in nanoscale through simple synthesis for boosting the conversion of CO_2_ and further, extending the homojunction family.

Here, we report a highly orientated phase-enabled MOF homojunction with a well-defined junction interface for visible-light-driven CO_2_ reduction. It is well known that the dynamic feature of supramolecular coordination bonds in MOFs allows metal nanoparticles (NPs) to react with MOFs^28,29^. Metal NPs can be either embedded into MOFs^30,31^ or loaded on the surface of MOFs^32^. Herein, we creatively developed a transition metal NPs-directed MOFs growth route to create, in one step, a MOF homojunction composed of two stacked concentric MOF nanoplates with the same orientation and uniform size. The nanoplates had analogous cobalt porphyrin composition but slightly different crystal structure and varied energy level alignments. Meanwhile, in situ surface photovoltage microscopy (SPVM) images^33–35^, obtained through integrating Kelvin probe force microscopy (KPFM)) with appropriate light illumination, visually and straightforwardly revealed the light-induced charge separation between the two stacked nanoplates in MOF nanostructures. The as-prepared MOF nanoplate-based homojunctions demonstrated ca. 2.5 times enhanced CO_2_ reduction to CO, over single MOF nanoplates, in water under visible light and high stability for at least 22 h. Importantly, the high selectivity (ca. 100%) of our system toward CO synthesis in water media surpassed the overwhelming majority of photocatalysts.

## Results

### Synthesis and characterization of Co-MOF

**Fig. 1:**
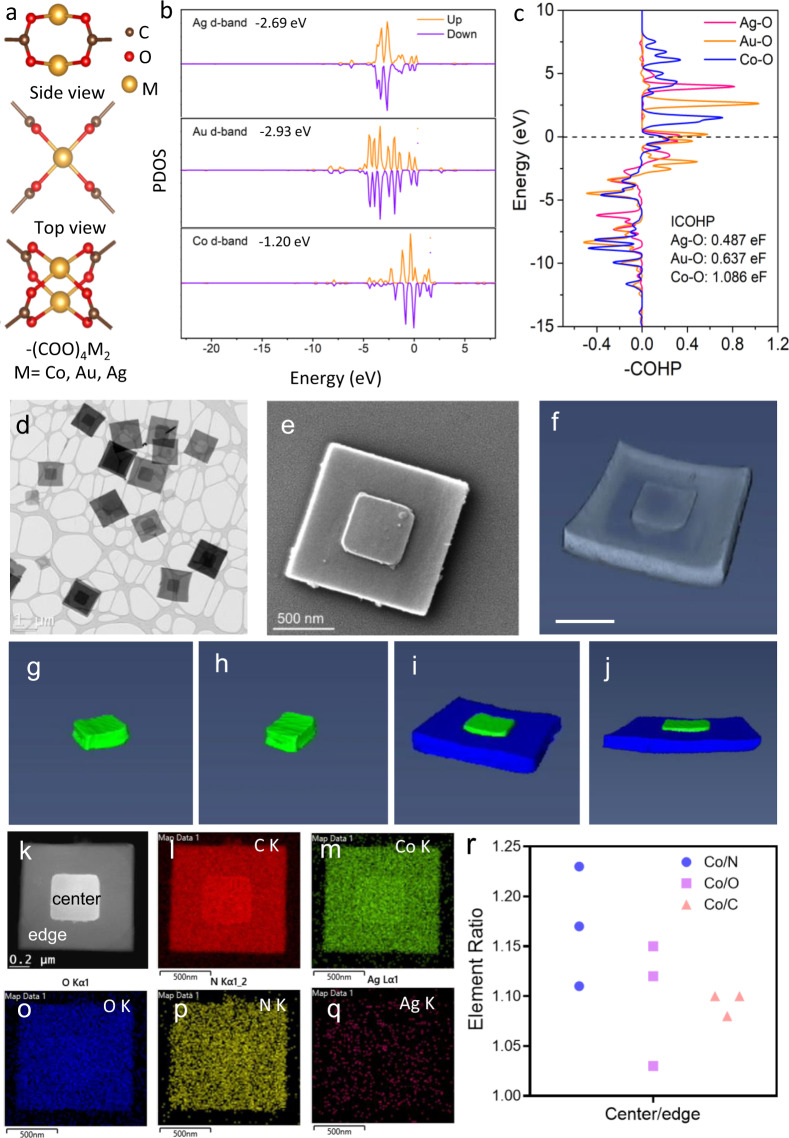
Structural and elemental analysis of Co-MOF-3 nanostacks. **a** Schematic illustrations of –(COO)_4_M_2_ (M=Co, Au, Ag). **b** PDOS of *d*-band of M in –(COO)_4_M_2_ (up: spin up, down: spin down) and corresponding caculated d-band center values. Ef = 0. **c** COHP bonding analysis of M–O interactions in –(COO)_4_M_2_. Ef = 0. The unit is eV/f.u.(eF), standing for eV per formula unit. ICOHP represents the intensities of COHP. **d** A typical TEM image of Co-MOF-3. **e** The SEM image of Co-MOF-3. **f** 3D TEM image of Co-MOF-3 nanostack and 3D electron tomography reconstruction model of the small squared plate (**g**, **h**) and large squared plate in Co-MOF-3 (**i**, **j**) shown in **f**. Scale bar, 500 nm. **k** HAADF image of Co-MOF-3 and the corresponding EDS elemental mapping images of **l** C, **m** Co, **o** O, **p** N and **q** Ag in Co-MOF-3. **r** EDS peak-area ratios of K series for Co/N, Co/O, and Co/C between the central (center) and the peripheral area (edge) of Co-MOF-3.

The dynamic feature of supramolecular coordination bonds in MOFs allows metal NPs to direct MOFs’ synthesis through metal node exchange. One of the determining factors of the node exchange consists in the relative strength of coordination bonds between metal nodes and organic linkers. Generally, stronger coordination bonds can gain a competitive edge over weaker coordination bonds^28,29^. In MOFs, metal ions interact with –COOH in the organic linker of tetrakis (4-carboxyphenyl) porphyrin (TCPP) in the most common form of –(COO)_4_M_2_ (M=Co, Au, Ag) (Fig. 1a). It is well known that the d-band position of transition metals plays an important role when metals interplay with oxygen (O) atoms or molecules^36^. Hence, density-functional theory (DFT) was used to investigate the d-band position of transition metals in Co-MOF, Ag-MOFs, and Au-MOFs. The d orbital projected densities of states of M in –(COO)_4_M_2_ are plotted in Fig. 1b. A higher *d*-band center (*E*_*d*_) of Co (−1.20 eV) in –(COO)_4_Co_2_ than Au (−2.93 eV) in –(COO)_4_Au_2_ and Ag (−2.69 eV) in –(COO)_4_Ag_2_ suggests a decrease in the filling of the (*d–σ*)* state and stronger bonding of Co–O than Au–O and Ag–O. Moreover, the crystal orbital Hamilton population (COHP) was taken to chop the total band-structure energy of –(COO)_4_M_2_ into specified M–O interactions (Fig. 1c). Co–O shows the highest intensity of integrated COHP (1.086 eF) than Ag–O (0.487 eF) and Au–O (0.637 eF), further revealing the stronger bonding of Co–O and less destabilized feature of –(COO)_4_Co_2_^37^. Thus we decided to use weaker-interacting, transition metal Au/Ag NPs as potential inducers. Further considering cost effectiveness, we designed hollow, instead of solid, Au/Ag nanocubes to induce the growth of cobalt MOFs in this work.

Hollow Au/Ag nanocubes were prepared following a previously reported method^38^. They were ca. 45 nm in size and had a narrow size distribution (Supplementary Fig. 1). From the TEM image (Supplementary Fig. 2), we can clearly see the hollow structure. X-ray photoelectron spectroscopy (XPS) (Supplementary Fig. 3) and element dispersive spectroscopy (EDS) (Supplementary Fig. 4) confirmed the co-existence of Au and Ag in the nanocubes and the Ag/Au ratio of 47:53. The preset amount of hollow Au/Ag nanocubes was then added into the MOF precursor solution to initiate the MOF synthesis reaction. As a result, three different samples, here named Co-MOF-1, Co-MOF-2, and Co-MOF-3, with different morphologies were formed by just adjusting the amount (1, 2, and 5 μmol) of hollow Au/Ag nanocubes. The red shift of the Söret band in the ultraviolet–visible (UV–vis) absorption spectra of three MOFs (Supplementary Fig. 5) suggested the formation of coordination polymers^39,40^. As shown in Supplementary Fig. 6, the Raman spectra of Co-MOFs are similar but quite different from that of the TCPP monomer ranging from 1500 to 1800 cm^−1^, suggesting the coordination of -COOH in TCPP with Co atoms in the Co-MOFs, and similar chemical bonding in Co-MOFs.

A typical TEM image of Co-MOF-1 (Supplementary Fig. 7) reveals its morphology, mainly single square nanoplates plus a small number of rectangular ones with their lateral size ranging from 200 to 300 nm, and its high-resolution TEM (HR-TEM) and a single set of selected-area electron diffraction (SAED) pattern suggested single crystal feature (Supplementary Fig. 8). After we increased the Ag/Au NPs to 2 μmol, a few stacked MOFs appeared in resultant Co-MOF-2, but most of the MOFs were still single unstacked small nanoplates (Supplementary Fig. 9). When the Ag/Au NPs reached 5 μmol, Co-MOF-3 with two well-stacked concentric-aligned MOF nanoplates with identical orientation were formed, as shown in Fig. 1d and Supplementary Fig. 10. Scanning electron microscopes (SEM) image (Fig. 1e) and 3D TEM image (Fig. 1f) confirmed the stacked structure and excluded the possibility of a core-shell structure. 3D topography reconstruction (Fig. 1g–j and Supplementary Movie 1) suggested that the smaller nanoplate was partially embedded into the large nanoplate. Such a well-defined unique structure is very rare in either heterojunctions or homojucntions, since it is still very challenging to control the relative orientations of the two components at their interface. An AFM image of Co-MOF-3 nanoplates implies that the lateral sizes were much larger than the heights of both large and small nanoplates (Supplementary Fig. 11). AFM (Supplementary Fig. 12) and SEM (Supplementary Fig. 13) images of a single Co-MOF-3 particle with the small nanoplate removed, showed a reduction in height ca. 27 nm in the center of the large nanoplate, suggesting that the smaller nanoplate was partially embedded into the large nanoplate, instead of merely stacking on the surface, thereby producing an intimate and robust junction interface between the two.

Moreover, a high-angle annular dark-field (HADDF) image of a representative Co-MOF-3 nanostack (Fig. 1k) and the corresponding EDS element mapping analysis of C (Fig. 1l), Co (Fig. 1m), O (Fig. 1o), N (Fig. 1p), and Ag (Fig. 1q) were conducted. The C, Co, O, and N elements were found to be evenly distributed in both large and small nanoplates in Co-MOF-3, suggesting a uniform Co-MOF composition. However, no detectable signal from Ag or Au elements indicates that the hollow Au/Ag nanocubes only acted as an inducer for the synthesis of MOF nanostacks, in line with EDS spectra (Supplementary Fig. 14). Meanwhile, the XPS spectrum of Co-MOF-3 (Supplementary Fig. 15) suggested the presence of Co element, but no apparent Au and Ag elements left in the samples. Besides, the ratios of integrated peaks of Co K series to N K series (Co/N), O K series (Co/O), and C K series (Co/C) from mapping images of different Co-MOF-3 nanoplates were calculated and are listed in Fig. 1r and Supplementary Table 1. It can be seen that the ratios of Co/N, Co/O, and Co/C in the central part of three randomly selected Co-MOF-3 nanostacks were consistently higher than those in the peripheral part, suggesting a slightly higher Co content in smaller nanoplates than large ones.

### Crystal structure, and optical and electronic properties of Co-MOF-3 nanostacks

**Fig. 2:**
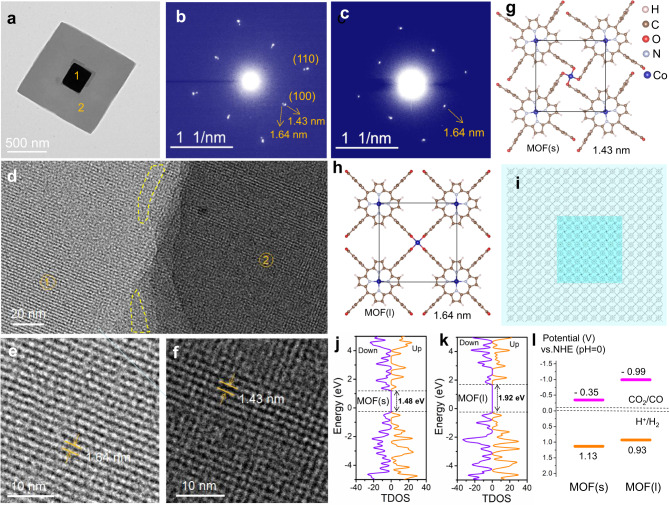
Crystal structure of Co-MOF-3 nanostacks. **a** TEM image and **b**, **c** the corresponding SAED from the central (position 1 in **a**) and peripheral (position 2 in **a**) areas of Co-MOF-3, respectively. **d** HR-TEM image of the epitaxial inteface between the central and peripheral parts (yellow polygons represent interfacial dislocation). **e**, **f** Enlarged lattice image of position 1 in **d** and position 2 in **d**, respectively. **g** Cystal structural model of the smaller MOF, named MOF(s), in nanostacked Co-MOF-3. **h** Cystal structural model of the larger MOF, named MOF(l). The rectangular boxes in **g** and **h** highlight the unit cells. **i** Proposed top-view structure of Co-MOF-3 nanostacks. **j**, **k** TDOS plots of monolayer MOF(s) and MOF(l) in nanostacks, respectively. **l** Calculated energy levels of monolayer MOF(s) and MOF(l) in nanostacks (Potential vs. NHE (pH = 0)).

The atomic structure of one typical Co-MOF-3 nanostack was investigated by SAED, as shown in Fig. 2a. The SAED patterns from the central part (Fig. 2b) and peripheral part (Fig. 2c) of nanostack. The fourfold symmetry diffraction spots indicate the tetragonal crystal structure of Co-MOF-3 nanoplates^4,40,41^. Interestingly, we observed two different interplanar distances of 1.64 and 1.43 nm for the (100) diffraction from the central part, while only one spacing of 1.64 nm from the peripheral part, suggesting their slightly different crystal structures of two nanoplates in one nanostack, and a similar phenomenon was noticed in epitaxial heterostructures^42^. From the HR-TEM image (Fig. 2d) of the interface between the central and peripheral parts, a certain dislocation at the interface was observed. The lattice fringes of a peripheral and central region near the interface were observed, and the lattice distances measured to be 1.64 nm (Fig. 2e) and 1.43 nm (Fig. 2f), respectively, in accord with the SAED results. Moreover, two sets of interplanar distances were also noticed for (110), (200), (210), (220) crystal lattice planes (Fig. 2e, f and Supplementary Fig. 16)^43,44^. Besides, the TEM image of one Co-MOF-3 nanoplate with a missing central part (Supplementary Fig. 17a, b, d–g) shows continuous crystal lattice fringes with the same distance, and the corresponding Fast Fourier Transform image (Supplementary Fig. 17c) exhibits only a single set of (100) diffraction spots. Furthermore, we have done extensive searching under TEM and finally found several small plates with a similar size of MOF(s). Their corresponding SAED (Supplementary Fig. 18) also agrees well with that of the MOF(s), suggesting that they may be the small plates falling down from the Co-MOF-3 stacks. All these observations further verified our proposed structure. All these data confirmed that two nanoplates in the Co-MOF-3 nanostacks had slightly different crystal cell parameters. Although we had also tried the XRD characterization of MOFs (Supplementary Fig. 19), unfortunately, the XRD patterns are noisy and not good enough to verify the crystal structure. There may be two reasons; one is that the long-range order in MOF or COF NPs is often not good as the bulk materials. The other possible explanation is that although the Ag/Au NPs and polyvinylpyrrolidone (PVP) in this system are very effective for regulating and controlling MOFs’ morphology, they inevitably decreased the crystalline degree of MOF NPs.

DFT simulations were conducted to reveal the crystal structure of two stacked nanoplates in Co-MOF-3, according to SAED diffractions^44,45^. The smaller MOF nanoplate, with lattice constants of *a* = *b* = 14.3 nm and *α* = *β* = *γ* = 90°, in the nanostacked Co-MOF-3 was named MOF(s), while the crystal structural model of the larger MOF nanoplate, with lattice constants of *a* = *b* = 16.4 nm and *α* = *β* = *γ* = 90°, was named MOF(l). Their crystal structural models are displayed in Fig. 2g, h, respectively. Figure 2i shows the proposed top-view structure of Co-MOF-3 nanostacks composed of MOF(s) and MOF(l). Moreover, the three-dimensional (3D) crystal structural models of MOF(l) and MOF(s) are shown in Supplementary Figs. 20–23. Considering the charge transfer mainly happens at the contact interface, the interfacial electronic structures of monolayer MOF(l) and MOF(s) were investigated using total densities of states (TDOS) (Fig. 2j, k), respectively^45^. Moreover, the bandgap of 2D MOF(s) and 2D MOF(l) was calculated to be 1.48 and 1.92 eV, respectively. The higher conduction band minimum (CBM, −0.99 eV) and lower valence band maximum (VBM, 0.93 eV) of MOF(l) than those of MOF(s) indicate the formation of the type-II homojunction (Fig. 2l). Thus, MOF(l) can transfer electrons to MOF(s) while holes transport in the opposite direction, so as to promote the charge separation in a photochemical reaction. Besides, the bandgap of 3D MOF(l) and MOF(s) are only a little smaller than corresponding 2D ones, and their relative band alignment is very similar (Supplementary Fig. 24). It should be noted that MOF(s) was partially embedded into MOF(l). Thus, photogenerated charges cannot only transport in the vertical direction but also the horizontal direction, as shown in Supplementary Fig. 25, which is rare for epitaxial heterojunctions and homojunctions^40^.

The experimental bandgap of Co-MOF-3 was determined to be 1.46 eV from UV–vis diffuse reflectance spectra and the Tauc plot (Supplementary Fig. 26), which is close to the calculated bandgap of 2D MOF(s) (1.28 eV). CV curves of Co-MOF-3 at a scan rate of 10 mV s^−1^ were further collected in acetonitrile and 0.1 M Tetrabutylammonium hexafluorophosphate (Supplementary Fig. 27), by using the Ferrocenium/ferrocene (*F*_*c*_/*F*_*c*+_) redox couple to calibrate the pseudo reference electrode^46^. From this experiment, the VBM and CBM were determined to be −0.55 and 0.91 eV, respectively, in line with DFT calculation results and suggesting that the energy band of Co-MOF-3 satisfies the requirement of CO_2_ reduction to CO.

**Fig. 3:**
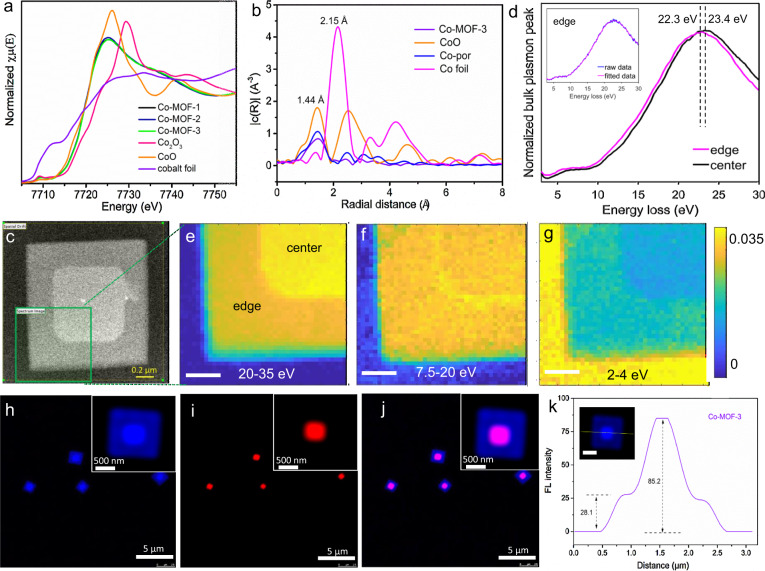
Elemental analysis of Co-MOF-3 nanostacks. **a** Normalized XANES spectra at Co *K*-edge for all the Co-MOFs and references of Co_2_O_3_, CoO, and Co foil. **b** Co *K*-edge Fourier transform *k*^2^-weighted EXAFS (not phase corrected) of Co-MOF-3, CoO, Co foil, and Co-porphyrin (Co-por). **c** HADDF image of of Co-MOF-3. **d** The low-loss EELS spectra of edge and center (after Gaussian smoothing). The inset is the raw data and Guassian fitting curve of EELS spectrum of edge. Low-loss EELS mapping in the range of **e** 20–35 eV, **f** 7.5–20 eV and **g** 2–4 eV. The low-loss EELS mapping was normalized by the integral signal, extracted with background removal, and smoothed by the Gaussian fitting model. Non-negative matrix factorization (NMF) was applied to the 41 × 41 pixels low-loss EELS spectra dataset. **h**–**j** Super-resolution multiphoton confocal images of Co-MOF-3. The excitation of wavelength is 405 nm and the emission was collected using (**h**) a 412–472-nm bandpass filter (blue) or (**i**) a 559–682-nm bandpass filter (red), respectively, and **j** merged image of **h** and **i**. **k** The FL intensity profile of the inset Co-MOF-3 image; the scale bar is 500 nm.

Moreover, synchrotron XAS was used to analyze the coordination geometry, the local chemical environment, and the metal valence. Figure 3a presents Co *K*-edge X-ray absorption near-edge structure (XANES) spectra of the Co-MOFs and references, including Co foil, CoO, and Co_3_O_4_. The pre-edge peak signal at 7712 eV of Co foil is not detected in Co-MOFs due to the fully occupied 3d orbital of Co^2+^, excluding the existence of Co (0) in Co-MOFs^47^. The absorption edge position at 7728 eV for Co-MOFs implies that the Co atoms in Co-MOFs are present as Co^2+^ (the same position as Co^2+^ in CoO), which is consistent with our proposed Co-MOF structure.

From the Fourier-transformed *k*^*2*^-weighted extended X-ray absorption fine structure (EXAFS) results (Fig. 3b), it can be seen that the Co-MOF-3 has a similar peak position with porphyrin Co coordination center (containing CoN_4_) and CoO at ca. 1.44 Å, which is mainly ascribed to the Co–N/O coordination at the first shell. Besides, there is neither the Co–Co interaction peak at 2.15 Å nor other high-shell peaks in the EXAFS spectra of Co-MOF-3, further confirming that no heavy backscattering atoms (Co) are connected to Co sites. Moreover, the local environment of the Co atoms was determined using the phase shift, and amplitude functions of Co–N contribution and Co–C contribution generated using MOF(s) structure, and the coordination number of Co atoms in Co-MOF-3 is found to be 5. It should be noted that it is very challenging to distinguish first-row ligands (carbon, nitrogen, oxygen, and fluorine) using EXAFS since the photoelectron scattering function is weakly dependent on the Z number. Therefore, we can conclude that the samples present approximately five neighbors in the first shell, but we cannot affirm which atoms (O or N) are present since they would give a similar result. Thus the pillar structure of Co-MOF-3 was confirmed.

Besides, the carbon K-edge core-loss mapping (Supplementary Fig. 28a–c) and spectra (Supplementary Fig. 28d) were obtained from electron energy loss spectroscopy (EELS)^48^. The stronger carbon element signal of raw data in the central part of position 1 than in the peripheral part of position 2 is mainly due to the center part’s relatively higher thickness (see 3D TEM reconstruction, AFM, and SEM images). To eliminate the effect of thickness, the carbon K-edge core-loss EELS image and spectra were normalized by the integral signal, extracted with background removal, and smoothed by the Gaussian fitting method. Normalized carbon *K*-edge from the core-loss EELS mapping and spectra are shown in Supplementary Fig 28e, f, and it is possible to observe the differences between the edges’ response intensity. Less carbon element signal was observed in the central part of position 1 than in the peripheral part of position 2, which suggested that the C content in the former is smaller than in the latter, in good agreement with our EDS elemental analysis in Fig. 1r. Besides, an energy loss-peak at 285 eV induced by transitions from 1*s* to π* molecular orbital in carbon due to the presence of *sp*^2^ carbon, and a transition from 1*s* to σ* in carbon at 295 eV^49^, were observed in both central and peripheral parts. It indicated a similar carbon environment in these two parts, consistent with our proposed structures.

Furthermore, the low-loss EELS spectra of Co-MOF-3 nanostack (Fig. 3c) were collected pixelwise (41 × 41 pixels) via using scanning transmission electron microscopy (STEM). Bulk plasmon bands of edge and center regions were extracted from the corresponding pixels in the raw dataset and then smoothed by the Gaussian method (Fig. 3d). As shown in the inset of Fig. 3d, the raw curve and fitting curve of edge EELS spectrum matched very well, indicative of a very-good fitting. The bulk plasmon peak positions of the edge and center regions were found slightly shifted, suggesting the difference of the valence electron density between MOF(s) in center and MOF(l) in center and edge in Co-MOF-3 nanostack. We further mapped the low-loss EELS dataset of Co-MOF-3 in 2–35-eV energy range by applying non-negative matrix factorization (NMF)^48^. Here, the 20–35 eV (Fig. 3e) and 7.5–20 eV (Fig. 3f) are both attributed to the bulk plasmon band, and the thickness effect was removed by the Fourier-log deconvolution method. It should be noted that although 2–4 eV (Fig. 3g) is due to the tail of the zero loss-peak not bulk plasmon, it supports the three components (center, edge, and substrate) in a whole STEM/EELS view. The different variation trend of relative intensity of two target components (center and edge) in these energy ranges straightforwardly confirmed center/edge difference in terms of valence electron density.

As shown in Supplementary Fig. 29, there are two peaks in the fluorescence (FL) spectrum of a single Co-MOF-3 nanostack excited at 450 nm. One is at 430–470 nm, which should be ascribed to triplet-triplet annihilation produced S2 emission (S2–S0), and the other is at 600–730 nm of S1–S0 emission. Furthermore, much lower intensity of S2 emission than S1 emission suggests the efficient charge transfer between porphyrins in MOFs. From Fig. 3h–j, Supplementary Fig. 30, and Supplementary Movie 2, we can see that the FL intensity for both S1 and S2 emission in the center is much higher than the edge part in the Co-MOF-3. To exclude the effect of thickness, we have quantitatively measured the FL intensity profile from one Co-MOF-3 nanostack. It was found that the intensity ratio of the center to edge in the nanostack reached ca. 85.2:28.1 (Fig. 3k), which is much higher than the thickness ratio (1:1.3–1.6) of center to edge in the Co-MOF-3 based on the AFM measurements and 3D reconstruction. Therefore, the FL intensity difference should not mainly result from the thickness difference. Higher FL intensity of MOF(s) than MOF(l) further suggested the difference in quenching rates attributed to differences in the free energies of electron transfer between porphyrin molecules in MOFs. A much remarkable quenching of red FL than blue FL in MOF(l) compared with MOF(s) suggested the more S1–S0 state quenching than S2–S0 in the former, which may be affected by the charge transfer between MOF(s) and MOF(l) at the junction and self-quenching properties of these two MOFs due to the different molecular arrangement.

### Formation mechanism of Co-MOF-3 nanostacks

**Fig. 4:**
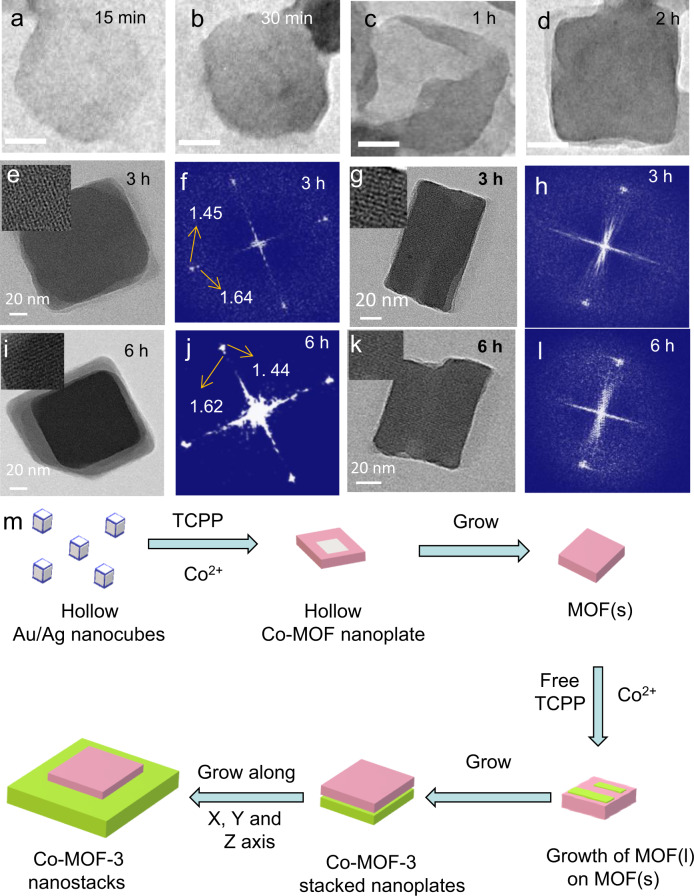
Morphological evolution of Co-MOF-3 nanostacks in time. TEM images of Co-MOF-3 at various reaction time of 15 min (**a**), 30 min (**b**), 1 h (**c**), and 2 h (**d**) (bar is 50 nm). TEM and HE-TEM images of top (**e**) and side (**g**) Co-MOF-3 at 3-h reaction time, and the corresponding FFT images of **f** and **h**, respectively. Top (**i**) and side (**k**) TEM and HR-TEM images of Co-MOF-3 at 6 h and corresponding FFT images of **j** and **l**, respectively. **m** Schematic diagram of the formation mechanism of Co-MOF-3 nanostacks directed by the hollow Au/Ag nanocubes.

The formation mechanism of asymmetric (in the thickness direction) Co-MOF-3 nanostacks was revealed by tracking intermediate evolution in time^50^. At the very beginning, organic porphyrin molecules are easily absorbed onto the surface of Au/Ag nanocubes through the interaction of Au/Ag with COO– or Au/Ag and π electron interaction in porphyrin and then prearrange through self-assembly^51^. The porphyrins are generally prearranged on the surface of Au or Ag as a form of inclined configuration with shorter center distance than free self-assembly due to restricted arrangement, and it is the same as what we have observed in MOF(s). The role of hollow Au/Ag nanocubes as self-assembly inducer was confirmed by more TEM observations of Co-MOF-3 nanostacks (Supplementary Figs. 31 and 32). Subsquently, primary hollow square-shaped Co-MOF nanoplates (Fig. 4a and Supplementary Fig. 34) were formed through metal exchange from Ag/Au to Co due to stronger bonding strength of Co–O than Au–O and Ag–O. It was supported by the computer simulation of *d*-band center and COHP of Co–O, Ag–O, and Au–O (Fig. 1b, c). And then primary solid MOF(s) nanoplates were formed after the small hollow Co-MOF nanoplates grew along lateral directions (Fig. 4b). Meanwhile, free TCPP molecules and Co^2+^ in the solution formed the second nanoplate of MOF(l) on the 001 facet of the primary nanoplate, which act as a substrate due to similar composition (Fig. 4c and Supplementary Fig. 34). Due to the crystal structure of MOF(l) is thermodynamically more stable than MOF(s), the growth speed of MOF(l) should be faster than MOF(s).

Thus, following the fast growth around the nucleation sites, the MOF(l) layer gradually grow on the (001) facet of the primary MOF(s) (Fig. 4d and Supplementary Fig. 35) at 2 h. Furthermore, it reached a similar lateral size and orientation with the primary layer at 3 h, confirmed by both the top view (Fig. 4e and Supplementary Fig. 36) and side view (Fig. 4g), and the corresponding TEM images of the two facets are shown in Fig. 4f, h, respectively. It was found that two sets of SAED diffraction spots already emerged (Fig. 4e–h). Considering the more thermodynamically stable crystal structure of MOF(l), the growth speed of MOF(l) should be faster than MOF(s). Subsequently, both layers continued to grow, mainly in two dimensions due to the attachment of PVP in (100) surface. For the 6-h sample, one nanolayer was already slightly larger than the other (Fig. 4i, k and Supplementary Fig. 37). Consistently, two sets of (100) diffraction spots were observed (inset in Fig. 4j, l). And the smaller layer was partially embedded into the large layer, likely resulting from different growth rates along the [001] axis of both nanoplates. As a result, Co-MOF-3 nanostacks with slightly different crystal phases were prepared. The schematic diagram of the formation mechanism was summarized in Fig. 4m, which was also supported by time-dependent UV–vis spectra of the reaction solutions (Supplementary Fig. 38). Thus, we named this method “transition metal NPs-directed MOF growth.” Moreover, when we increase the amount of Au/Ag to 15 μmol, the homojunction still can be formed (Supplementary Fig. 39), but a larger diameter of MOF(s) in Co-MOF nanostacks can be observed, which is in line with our proposed mechanism.

Furthermore, we have tuned the ratio of Au/Ag in transition metal NPs and investigated the formation of nanostacks. First, we prepared the hollow Au/Ag nanocubes with an Ag/Au molar ratio of 72:28 (Supplementary Fig. 40), different from that (Ag/Au molar ratio of 47:53) shown in our initial manuscript, and used them to induce Co-MOF formation. Once again, stacked nanoplates with two SAED diffraction sets were observed (Supplementary Fig. 41). We then further increased the Ag/Au ratio of Au/Ag nanocubes to 89:11, MOF nanostacks can also be successfully obtained (Supplementary Figs. 42, 43). In two extreme cases, we used pure metals, the solid Ag nanocubes (Supplementary Fig. 44) and solid Au NPs (Supplementary Fig. 46), as inductors and in both cases, the stacked nanoplates with two sets of SAED diffraction were observed (Supplementary Figs. 45,  47–49), similar to the use of hollow alloy NPs. We can already reasonably conclude that our method for the synthesis of Co-MOF nanostacks is universal.

### SPVM characterization of Co-MOF-3 homojunction

**Fig. 5:**
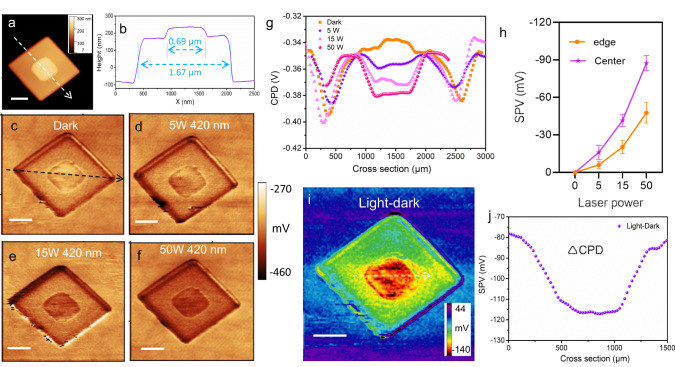
SPVM analyses of stacked phase-enabled Co-MOF-3 homojunction. **a** AFM topography image and **b** the corresponding height profile of Co-MOF-3. **c** KPFM image of Co-MOF-3 under no illumination (dark condition). KPFM image of Co-MOF-3 under the irradiation of 420-nm LED light with the power of **d** 5 W, **e** 15 W, and **f** 50 W. **g** The corresponding normalized CPD profiles across the center of Co-MOF-3 (see the arrow line in **c**) under the dark and irradiation conditions. **h** The statistical SPV values (*n* = 5, SPV, ΔCPD = CPD_light_ − CPD_dark_) collected from the center (purple) and peripheral (orange) regions, respectively, in the (001) facet of Co-MOF-3 stacked nanoplate under the illumination with various power densities (correction of SPV values was done by subtracting the corresponding CPD vaules of the HOPG support). Data are presented as mean values ±S.D. **i** SPVM image obtained by subtracting the potential under the dark condition from that under 50-W illumination and **j** the corresponding SPV profile.

We adopted SPVM technique^33,34^ to visualize the spatial charge transfer and separation between the two stacked nanoplates in Co-MOF-3 by integrating light illumination with the KPFM to map the surface photovoltage (SPV). The AFM topography image of one typical Co-MOF-3 nanostack is shown in Fig. 5a and its corresponding height profile in Fig. 5b. The edge lengths of two nanoplates were 0.69 and 1.67 μm, respectively. When the measurement was done without illumination, the Fermi levels of MOF(s) and MOF(l) were aligned at a thermal equilibrium state. Therefore, the contact potential difference (CPD) represents the local vacuum energy level variations relative to Fermi level positions at phase junction. The CPD of MOF(l) is about 15 mV lower than that of MOF(s), suggesting that the work function of MOF(l) is 15 eV higher than that of MOF(s). Thus, it can be deduced the formation of an internal built-in electric field and an upward band bending from MOF(s) to MOF(l) across the interface. It suggested that photogenerated electrons could transfer from MOF(l) to MOF(s) across the phase junction interface, according to the surface potential and the direction of the built-in electric field^52^.

The CPD curves across the center and the peripheral parts were profiled from the KPFM images of Co-MOF-3 under the dark condition (Fig. 5c) and the irradiation of a 420-nm LED with radiation power of 5 W (Fig. 4d), 15 W (Fig. 5e), and 50 W (Fig. 5f), respectively. Under the dark condition, the CPD values of the small nanoplate (MOF(s)) were lower than those of the large one (MOF(l)) in the Co-MOF-3 nanostack, suggesting a higher work function of the smaller nanoplate^35^. To investigate the impact of illumination, a LED (420 nm) lamp with tunable power was applied in the direction parallel to the (001) facet of the Co-MOF-3 nanostack. It was found that the CPD values decrease with increasing light intensity, thus the decrease of CPD yields a negative SPV (Fig. 5g). However, the increase of MOF(s) was more significant, increasing the CPD difference between the smaller and large nanoplates. In other words, the ΔCPD values of the MOF(s) became higher than those of MOF(l) under illumination.

The SPV values, obtained from five sets of data and after subtraction of the background CPD values, are shown in Fig. 5h. Moreover, the SPV image (SPV, ΔCPD = CPD_light_ − CPD_dark_, Fig. 5i) of the nanostack was constructed by subtracting the KPFM image in the dark from the image (Fig. 5c) under illumination (Fig. 5f), and the corresponding SPV profile is shown in Fig. 5j. A remarkably higher SPV value of ca. 40 mV was observed for MOF(s) nanoplate. Combining the results of direction of the built-in electric field and calculated relative energy band alignment of MOF(l) and MOF(s), we infer that the most possible reason for this phenomenon could be charge separation and transfer between homojunction in Co-MOF-3^53,54^. To the best of our knowledge, it is the first report on MOF-based homojunction, innovatively demonstrated by the SPVM method.

The charge-carrier dynamic of Co-MOF-3 nanostacks is revealed by steady-state FL (Supplementary Fig. 50a) and time-resolved FL characterizations (Supplementary Fig. 50b). The FL spectrum of the Co-MOF-3 nanostacks presented a more remarkable FL quenching with respect to Co-MOF-1 and Co-MOF-2. Along with the shorter average lifetime (*τ*_avg_) of Co-MOF-3 (2.74 ns) than those of Co-MOF-2 (4.05 ns) and Co-MOF-1 (10.74 ns), it could be reasonably concluded that the increase of transfer and separation efficiency of charge carriers in Co-MOF-3 resulted from the short charge-transport distance, large interfacial contact area (both side-to-side and face-to-face), and strong interfacial bonding in the as-prepared homojunction^25^. Moreover, the Co-MOF-3 nanostacks manifested the smallest semicircle in the Nyquist plots (Supplementary Fig. 51), highest transient photocurrent (Supplementary Fig. 52), and most significant amplitude of open circuit potential (OCP) values (Supplementary Fig. 53) under irradiation among the three Co-MOFs, further supporting the most efficient photoinduced electron-hole transport and separation of Co-MOF-3 nanostacks due to the structural homojunction.

### Photocatalytic CO_2_ reduction performance of Co-MOF-3 homojunction

**Fig. 6:**
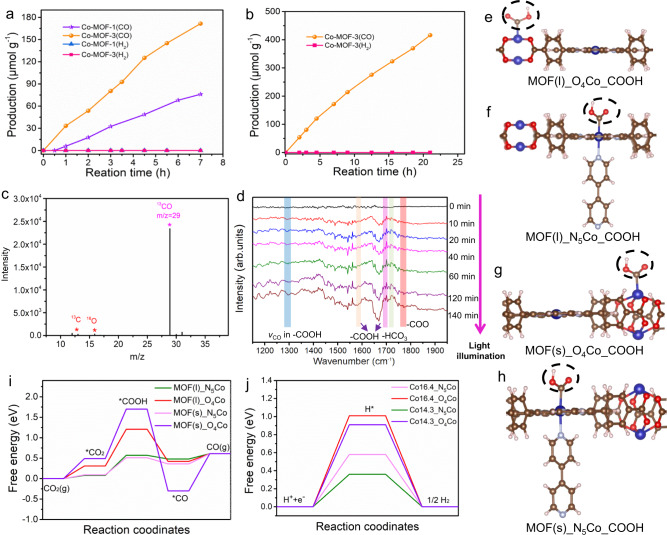
Photocatalytic CO_2_ reduction performance of Co-MOF-3 homojunction. **a** Evolution of CO and H_2_ as a function of reaction time during the CO_2_ reduction in aqueous solution under visible-light illumination for the Co-MOF-1 and Co-MOF-3 photocatalysts. **b** Stability of Co-MOF-3 during the continuous CO_2_ reduction reaction. **c** Isotope labeling of Co-MOF-3 system carried out by using ^13^CO_2_ gas. **d** In situ DRIFTS measurement of Co-MOF-3. **e**–**h** Schematic illustration of intermediate *COOH adsorbed onto MOF(l)_N_5_Co, MOF(l)_O_4_Co, MOF(s)_N_5_Co, and MOF(s)_O_4_Co in the Co-MOF-3 homojunction during CO_2_ reduction, respectively. Calculated free energy (Δ*G*) diagram of **i** CO_2_ reduction to CO and **j** HER on Co sites in varied units of MOF(l)_N_5_Co, MOF(l)_O_4_Co, MOF(s)_N_5_Co, and MOF(s)_O_4_Co, respectively (*U* = 0).

The photocatalytic CO_2_ reduction performance of the Co-MOF-3 (mainly composed of homojunction nanostacks) with Co-MOF-1 (mainly composed of single nanoplates) as control was evaluated in aqueous solution under visible-light irradiation (*λ* ≥ 400 nm, 1-atm CO_2_). Figure 6a shows that CO_2_ was light reduced by Co-MOF-1 to CO with a generation rate of 10.7 μmol h^−1^ g^−1^, which is close to the performance of Zn-porphyrin MOFs reported^54^. As expected, the Co-MOF-3 with the homojunction presented a much higher CO production rate of 27.1 μmol h^−1^ g^−1^, which is ca. 2.5 times higher than that of the Co-MOF-1. We believe that the increase in performance is mainly due to the promoted charge separation driven by the presence of the homojunction. Such a CO_2_ to CO conversion rate is moderate in the family of MOFs^55^, but higher than the rate of pure g-C_3_N_4_ (10.3 µmol h^−1^ g^−1^)^56^ and that of most Z-scheme heterojunctions^16^. No evident decrease of CO evolution rate was perceived after 22-h photocatalytic reaction (Fig. 6b), and no significant change of structure reflecting the high stability of the Co-MOF-3 as a photocatalyst. It should be noted that we did not use organic solvent of acetonitrile, which was used in most photocatalytic CO_2_ reduction to lift the efficiency of photocatalytic CO_2_ reduction and contributing to the stability of photocatalysts. More importantly, no H_2_ or liquid products could be detected in this reaction system, indicating a superior selectivity (~100) of Co-MOF-3 toward CO production. Such a high CO selectivity of our Co-MOF systems should be closely related to the chemical composition and pore structure of MOFs^6,7,14^. Till now, among numerous literature reports on CO_2_ reduction, only a very few systems could achieve 100% selectivity for CO^19,21^.

The control experiments with inert gas (Ar), without light irradiation and in the absence of catalyst of Co-MOF-3 were carried out, and it was found that no CO or other products were detected during these processes (Supplementary Fig. 54). To further validate the origin of CO product, the ^13^CO_2_ isotope labeling experiment was further carried out^57,58^. As shown in Supplementary Fig. 55, the total ion chromatographic peaks at ca. 6.0 and ca. 15.8 min can be assigned to the CO (inset of Supplementary Fig. 55) and CO_2_, respectively. The mass spectrum (MS) with the signal peaks at *m/z* = 45 and *m/z* = 29 (^13^CO), *m/z* = 16 (^16^O) and *m/z* = 13(^13^C) from gas CO_2_ (ca. 15 min) confirmed the successful labeling (Fig. 6c and Supplementary Fig. 56). MS with the signal peak at *m/z* = 29 (^13^CO), from gas at ca. 6.0 min unambiguously indicated that ^13^CO indeed originated from light-driven ^13^CO_2_ reduction during the reaction. Moreover, in situ diffuse reflectance infrared Fourier transform spectroscopy (DRIFTS) suggested that the primary intermediates of CO_2_ photoreduction in Co-MOF-3 are the –COO and –COOH species (Fig. 6d)^58,59^.

The mechanism of the CO_2_ to CO conversion in the Co-MOF-3 homojunction system was investigated using DFT. It is well known that the photocatalytic reduction depends on the energy level of photocatalysts and the adsorption and desorption energies of the starting material, intermediate, and the product. Generally, the CO_2_-to-CO reduction reaction includes the following four steps: (i) the CO_2_ adsorption onto catalysts to form *CO_2_, (ii) the H^+^ and e^−^ coupled transfer to *CO_2_ and producing an intermediate of *COOH, (iii) second electron and proton transfer to *COOH and generating *CO and H_2_O, (iv) the desorption of *CO from catalysts to release the product of CO^22^. For porphyrins or analogs, the metal centers were demonstrated to be the preferably active sites as compared to C, N, and O elements in the porphyrin skeleton^60^. For our Co-MOF-3 homojunction, there are four different possible active sites of MOF(l)_N_5_Co, MOF(l)_O_4_Co, MOF(s)_N_5_Co, and MOF(s)_O_4_Co, and the schematic illustration of examples of intermediate *COOH adsorbed onto them are shown in Fig. 6i. Theoretical calculations showed that the N_5_Co presents a lower energy barrier in the path of *COOH and *CO intermediates than O_4_Co for either central MOF(s) or peripheral MOF(l) in Co-MOF-3 homojunction (Fig. 6j). It indicates that the CO_2_ reduction tends to occur on the N_4_Co sites. Meanwhile, because it protects O_4_Co as the node and linker of MOFs from damage in the process of catalysis, the photocatalytic durability of Co-MOF-3 is thus achieved. Besides, the CO_2_ adsorption energies onto the N_5_Co sites are quite small (ca. 0.08 eV), suggesting good adsorption capacility of Co-MOF-3. Meanwhile, the required H^+^ transfer energy from an intermediate of *CO_2_ to *COOH is about 0.42 eV over MOF(s)_N_5_Co, which is much smaller than the energy barrier of H^+^ reduction to H_2_ (0.87 eV, Fig. 6e), which well explains the observed high CO selectivity for Co-MOF-3 over the hydrogen evolution reaction.

## Discussion

In summary, we report a “transition metal NPs-directed MOF growth” process to construct well-defined MOF NPs-based homojunctions for visible-light-driven CO_2_ reduction. The as-prepared MOF homojunction was composed of two nanoplates with the (001) facet stacked together, and the smaller nanoplate was partially embedded into the large one to form a reliable homojunction interface. The two constituent nanoplates showed slightly different chemical compositions and crystal structures, and thereby different energy levels. This Janus structure displayed visible-light-driven CO_2_ reduction to CO in water with enhancement factor of 2.5 as compared with the case of single nanoplates, due to the formation of the unique homojunction of the hierarchical MOF NPs. It also exhibited excellent stability for at least 22 h during the continuous photocatalytic reaction. Most importantly, the CO selectivity of the as-prepared MOF was exceptional (ca. 100%). In situ SPVM was used to monitor the photogenerated charge flow direction in single MOF nanostacks and proved the enhanced charge separation due to the formation of the homojunction. The promoted separation of photogenerated charge carriers was verified by time-resolved FL lifetime and electrochemical impedance tests. We believe that the construction of MOF NPs-based homojunction is a very promising way to expand the MOF applicability to other photocatalytic and electrocatalytic reactions, such as water splitting and N_2_ fixation, and this work also offers a better understanding of the formation mechanism of MOF NPs.

## Methods

### Synthesis of Au/Ag nanocubes

The first step of synthesizing hollow Au/Ag nanocubes is the synthesis of cubical Ag templates. Ag nanocubes were prepared using a sulfide-mediated polyol method^38^, in which Ag^+^ was reduced to Ag^0^ by ethylene glycol in the presence of PVP and Na_2_S. Typically, 6 mL of ethylene glycol and 70 µL of Na_2_S·9H_2_O (3 mM) were added to the 20-mL vial and heated to 150 °C by using silicon oil under magnetic stirring. 1.5 mL of PVP (8 M) and 0.5 mL of Ag^+^ (280 mM) were added into the above solution quickly after 10 min. The solution was left for another 15 min. The color of the solution changed from colorless to green ochre. Centrifugation and washing of the NPs were repeated three times. Then Ag nanocubes were dispersed in water for the next step of Au/Ag nanocube synthesis. The obtention of hollow Au/Ag particles was performed by adding 5 mL of PVP (0.1%) and a magnetic stirrer in a round-bottom flask^36^, and then, 1 mL of Ag nanocube dispersion was added into the flask. The solution was heated until a mild boiling condition. The solution was left for 10 min under continuous stirring once the water vapor was condensed in the flask.

Then, the HAuCl_4_ (10 mM) solution was added using a syringe pump, and it was modified depending on the Ag:Au ratio. These volumes were 8.5, 16, and 27 mL of HAuCl_4_ (10 mM) from the lowest to the highest ratio, respectively. After the galvanic replacement process, the solution was centrifuged once at 9000 *g* for 30 min. The supernatant was removed, and the pellet was re-dispersed in 4 mL of ethanol and stored in a glass vial at 4 °C.

### Synthesis of round Au NPs

First, 10 mL of an aqueous solution containing 0.1-M HAuCl_4_ and 0.1-M PVP was prepared. Then 0.5 mL of NaBH_4_ solution (0.02 M) was added into it at 70 °C^45^. After 1-h reaction, Au NPs were obtained, followed by three times centrifugation/washing using pure water and finally stored in ethanol.

### Synthesis of Co-MOF-1, Co-MOF-2, and Co-MOF-3

At first, 1 mg of 2,4-dipyridine, 4.4-mg Co(NO_3_)_2_ 6H_2_O, and 10 mg of PVP (molecular weight: 44 kD) were dissolved in 4 mL of a DMF/ethanol mixture solution (DMF: ethanol= 3:1 in volume) in a 20-mL vial. Then TCPP (4 mg) and various amounts of Au/Ag nanocubes in 2 mL of DMF/ethanol (3:1 in volume) mixture solution were added and the mixture was ultrasonicated for 10 min. Afterward, it was sealed and placed into an 80 °C oven for 24 h. Co-MOF-1, Co-MOF-2, and Co-MOF-3 with different morphologies were formed with the addition of hollow Au/Ag nanocubes at 1, 2, and 5 μmol, respectively. To clarify the Co-MOF-3’s growth process, we also performed experiments with different reaction times of 15 min, 0.5, 1, 2, 3, and 6 h. Centrifugations/dispersions were repeated three times for purification. At last, the Co-MOF samples were kept in ethanol solution at 4 °C.

#### DFT calculation

The crystal structure of –(COO)_4_M_2_ (M = Co, Ag, Au) and Co-MOF-3 was optimized within the framework of DFT implemented in the Vienna ab initio Simulation Package code. The general gradient approximation plus U and Perdew–Burke–Ernzerhof exchange-correlation functional was used for structural optimization. The Van der Waals force correction was taken into account in using the VDW 11 scheme. The plane-wave cutoff energy was 450 eV. The convergence threshold for energy was 10^−5^ eV/cell. All calculations have been performed using the spin-polarized setup. Vacuum layers of 16 Å were introduced for the 2D monolayer slab. Lobster software was used to analyze the COHP plots of M–O in –(COO)_4_M_2_. The TDOS, Fermi energy, and vacuum energy level of MOF(s) and MOF(l) were obtained based on Heyd–Scuseria–Ernzerhof 06 hybrid exchange-correlation functional. The free energy calculation was thermal corrected at 298.15 K.

### Photocatalytic CO_2_ reduction

Photocatalytic CO_2_ reduction experiment was carried out in a 100-mL reaction vessel irradiated by a 300-W Xe lamp equipped with a 400-nm cutoff filter at ambient temperature. Typically, a water and TEOA solution (25 ml, v/v = 3:1) containing 2 mg of Co-MOF photocatalysts and [Ru-(bpy)_3_]Cl_2_ (500 μM) were purged with CO_2_ (purity ≥ 99.99%) for 30 min before irradiation. The temperature was held constant at 5 °C using a homeothermic cooling circulation pump. A gas chromatograph (Shimadzu GC-2014C, argon as carrier gas) equipped with a flame ionization detector was used to detect the CO and CH_4_ products, and a thermal conductivity detector was used to detect the formation of H_2_. Liquid products were detected using nuclear magnetic resonance with 600 MHz.

### FL measurements of Co-MOFs

The steady-state FL spectra of Co-MOF-1, Co-MOF-2, and Co-MOF-3 in ethanol solutions with the same Co-MOF concentration were recorded on a Fluorolog-3 system (Horiba Jobin Yvon) using a photomultiplier tube detector and under excitation of 430 nm. Lifetime measurements were carried out with the same excitation wavelength and at the emission wavelength of 650 nm using a time-resolved FL spectro-fluorometer (USA/CAN Photon Technology International Int.). Instrument response function was monitored to confirm the effectiveness of the measured FL lifetimes. The lifetimes of Co-MOFs were estimated through a double-exponential fitting. The average lifetimes were calculated according to the equation of *τ*_avg_ = (*A*_1_*τ*_1_ + *A*_2_*τ*_2_)/(*A*_1_ + *A*_2_), where *τ*_1_ and *τ*_2_ are FL lifetime components, and *A*_1_ and *A*_2_ are the corresponding fractions of *τ*_1_ and *τ*_2_.

### KPFM measurements

KPFM was performed using a conductive AFM Enviroscope system (Veeco Instruments, now Bruker) in a tapping mode at room temperature, equipped with a magnetic Co/Cr-coated tip (MESP-V2, Bruker) with a radius at the apex below 30 nm). We used a the double-pass technique, mechanically oscillating the cantilever at its first (fundamental) resonance (between 75 and 85 kHz, tip-dependent) for topography imaging in the first pass, and electrostatically oscillating the cantilever at the same frequency in the second pass while tracing the topography at a lift height of 30 nm. The amplitude of the AC excitation applied was 1 V. And the Co-MOF-3 dispersion samples were dropped onto conductive HOPG support. The LED laser powers of 5, 15, 50 W were used to irradiate the samples. The direction of light irradiation was almost parallel to the HOPG plate.

### XAS characterization of Co-MOFs

XANES and EXAFS were measured at the beamline 20-ID-C of the Advanced Photon Source at Argonne National Laboratory. A Si (111) double-crystal monochromator and focused beam (beam footprint on the sample of ∼500 μm (*v*) × 1000 μm (*h*)) were used to perform the measurements at Co *K*-edge (7709 eV). Harmonic rejection was facilitated by detuning 15% of the beam intensity at 500 eV above the edge of interest. Data were collected in transmission mode using ionization chambers filled with He. The sample was spread and sealed using Kapton^®^ tape, and the surface was tilted 45° to the incoming beam. EXAFS oscillations were extracted using Athena code and analyzed using Arthemis software. The local environments of the Co atoms were determined using the phase shift and amplitude functions of Co–N contribution and Co–C contribution generated using MOF(s) structure.

### Low-loss and core-loss EELS

TEM measurements were performed with JEOL JEM-2100 PLUS at 200 kV equipped with an in-column energy filter providing an energy resolution better than 150 meV. EELS image analysis was performed programmatically, using a code implemented in Matlab and HyperSpy module in Python. The carbon K-edge core-loss EELS spectra were normalized by the integral signal, extracted with background removal, and smoothed by the Gaussian fitting method^48^. The low-loss EELS spectra were normalized by the integral signal, extracted with background removal, and smoothed by the Gaussian fitting model. NMF was applied to the low-loss EELS spectrum image^61,62^. R-square is larger than 0.99 for all the fittings.

### In situ DRIFTS measurement

The DRIFTS measurements were performed by a FT-IR spectrometer (Nicolet 6700 Thermo Scientific, USA) with a designed reaction cell shown schematically in Fig. S58. A thin layer of Co-MOF-3 sample was placed onto  the substrate in the center of the designed setup. All the gases were pumped out from the setup using an ultra-high vacuum pump. Then CO_2_ gases were pumped in the setup to construct a CO_2_ atmosphere for CO_2_ photoreduction. Subsequently, the IR signal was in situ gathered through MCT detector during the reaction under visible light irradiation.

### Confocal FL imaging

Super-resolution multiphoton confocal image of Co-MOF-3 was acquired in TCS SP8 STED 3X (Leica) with the excitation wavelength of 405 nm from a Diode laser (50 mW). The emission was collected using a 412–472-nm bandpass filter (blue) or a 559–682-nm bandpass filter (red). Images were taken at a resolution of 1024 × 1024 pixels.

### 3D electron tomography reconstruction

The 3D tomography analysis was performed using Talos F200X G2. The specimens for 3D tomography observation were cylinders with 0.7 mm in diameter and 5 mm in height. The images of projection were captured at an interval of 0.11° over a total range of 180°. The exposure time was 2500 ms. The voxel resolution used in this measurement is 0.7 μm, which is applicable to the observation of SAMPLES with the size more than 1 μm. The data files containing three-dimensional information were processed using Dragonfly software, including image segmentation and 3D mesh reconstruction of different phases.

### Isotope labeling measurement

The isotope labeling measurement was carried out by using ^13^CO_2_ gas (Isotope purity, 99.9% and chemical purity, 99.9%, Beijing Innochem Technology Co., LTD.) as the carbon source with the same reaction set as mentioned above and the gas products were analyzed by gas chromatography–mass spectrometry (GC-2010, SHIMADAZU(CHINA) Co., LTD.) equipped with a column for detecting the products of ^13^CO (CP-Molsieve 5A.M, 25 m × 0.25 mm × 30 μm, SHIMADAZU (CHINA) Co., LTD.).

### Reporting summary

Further information on research design is available in the Nature Research Reporting Summary linked to this article.

## Supplementary information

Supplementary Information

Supplementary Movie 1

Supplementary Movie 2

Description of Additional Supplementary Files

## Data Availability

The authors declare that the main data supporting the findings of this study are available within the article and its Supplementary Information files. Extra data are available from the corresponding author upon request.

## Code availability

MATLAB codes used for analysis in this paper are available at https://github.com/wanting-rose/NMF-EELS-AnalysisOutline.git.
