# Supplementary material for: Phase-enabled metal-organic framework homojunction for highly selective CO2 photoreduction
Source: Nat Commun. 2021 Feb 23;12:1231. doi: 10.1038/s41467-021-21401-2 (PMC7902628; doi:10.1038/s41467-021-21401-2)
Supplement: Supplementary file 1 — Supplementary Information [file 41467_2021_21401_MOESM1_ESM.pdf]

Supplementary Information

# Phase-Enabled Metal-Organic Framework Homojunction for Highly Selective $CO_2$ Photoreduction

**Liu *et al.***

## 1. Supplementary Method

**Morphology and elemental characterization**. TEM, SEM and AFM were used to reveal the morphology of samples**.** The EDS analysis and SAED were also conducted in the TEM system to identify the distribution of chemical elements and crystal orientation of Co-MOFs and NPs. TEM experiments were performed in a JEOL JEM-2100F instrument at a voltage of 200 kV. TEM samples were dropped onto lacey carbon-coated copper grids. The SEM observations were carried out in SEM Tescan LYRA 3 equipment. Moreover, SEM samples were dropped onto polished silicon wafers. Then the topography of Co-MOF samples was characterized on a MultiMode 8 AFM (Bruker) in a tapping mode at room temperature. The Co-MOF-3 ethanol dispersion was dropped on to the cleaved mica surface and dried naturally. The corresponding height and lateral size measurements Co-MOF-3 samples were conducted using software of Nanoscope analysis 1.5.

**Electrochemical measurements of Co-MOFs.** Electrochemical measurements of Co-MOFs were carried out on a CHI 660C electrochemical workstation (CH Instruments) using a standard three-electrode configuration (the counter electrode: Pt wire, the reference electrode: Ag/AgCl and work electrode: fluorine-doped tin oxide (FTO) covered glass, coated with Co-MOF samples). 0.5 M $Na_2SO_4$ (pH = 6.8) aqueous solution was used as the electrolyte, $O_2$ was removed by $N_2$ bubbling into electrolyte more than 30 min. Impedance curves were collected from 100 kHz to 10 MHz with a bias voltage of 200 mV. Photocurrent experiments were performed in the dark or under an AM1.5G solar simulator (LCS-100, Newport) with the irradiation density of 100 mW/cm$^2$. Open circuit voltage was determined in the dark and under one sun solar light irradiation.

## 2. Supplementary Notes

To elucidate the role of the hollow Ag/Au nanocubes in the synthesis of Co-MOF-3, we investigated many Co-MOF-3 nanostacks with TEM techniques, and we were able to observe in completed Ag/Au nanocubes or Ag/Au NPs in a few Co-MOF-3 nanoplates. As shown in Supplementary Fig. 21, several small solid NPs with diameters smaller than 15 nm and a broken small nanocube with the equivalent size of hollow Ag/Au nanocubes (marked by a yellow square in Supplementary Fig. 21d) existed in the central part of Co-MOF-3, but none was observed in the peripheral part, evident from the contrast in HAADF images. The EDS spectra of the NPs and the broken small nanoplates clearly detected the signal of Ag and Au elements (Supplementary Fig. 22).

**Proposed formation mechanism of Co-MOF-3 nanostacks**

The whole proposed formation mechanism of different structures between center and lateral regions in Co-MOF-3 was revealed by tracking intermediate evolution in time using TEM and UV monitor, and computer simulation.

At the very beginning, organic porphyrin molecules are easily absorbed on the surface of Au/Ag nanocubes through Au/Ag and $COO^-$ or Au/Ag and π electron interaction in porphyrin and then pre-arrange through self-assembly. Such a phenomenon of porphyrin has been extensively studied in literature[1-8], often called restricted surface self-assembly. Moreover, in most of the cases, the porphyrins are pre-arranged on the surface of Au or Ag as a form of inclined configuration with shorter center distance than free self-assembly due to restricted arrangement, and it is the same as what we have observed in MOF(s). The role of hollow Au/Ag nanocubes as self-assembly inducer was confirmed by more TEM observations of Co-MOF-3 nanostacks (Supplementary Fig. 31,32).

Subsquently, primary hollow square-shaped Co-MOF nanoplates (Fig. 4a and Supplementary Fig.34) were formed through metal exchange from Ag/Au to Co due to stronger bonding strength of Co-O than Au-O and Ag-O.

It was supported by the computer simulation of d-band center and crystal orbital Hamilton population (COHP) of Co-O, Ag-O and Au-O[9]. As shown in Fig 1b, a higher d-band center ($E_d$) of Co (-1.20 eV) in -$(COO)_4Co_2$ than Au (-2.93 eV) in -$(COO)_4Au_2$ and Ag (-2.69 eV) in -$(COO)_4Ag_2$ suggests a decrease in filling of the (d-σ)* state and stronger bonding of Co-O than Au-O and Ag-O. Besides, Co-O shows the highest intensity of integrated COHP (1.086 eF) than Ag-O (0.487 eF) and Au-O (0.637 eF), further revealing the stronger bonding of Co-O and more stabilized feature of -$(COO)_4Co_2$ (Fig 1c).

And then, primary solid MOF(s) nanoplates were formed after the nucleating agent and template of small hollow Co-MOF nanoplates grew along with lateral directions (Fig. 4b). It should be noted that the polyvinyl pyrrolidone (PVP) ligand in this system tends to attach onto the (001) facet, rendering a growth speed perpendicular to the (001) facet much slower than that parallel to it[10]. Meanwhile, free TCPP molecules and $Co^{2+}$ in the solution formed the second nanoplate of MOF(l) on the 001 facet of the primary nanoplate, which acts as a substrate due to similar composition (Fig 4c and Supplementary Fig.34). Unlike MOF(l), the nucleator of MOF(s) is formed due to restricted self-assembly on Au/Ag surface. Thus it did not reach the most thermodynamically stable state, which was confirmed by DFT calculation of ca. 34.1 eV higher cohesive energy of MOF(l) (633.4 eV, PBE fromVASP) than MOF(s) (599.3 eV, PBE fromVASP). However, the differential energy value is still small comparing with the total bulk cohesive energy of MOF(l) and MOF(s), as may explain why the MOF(s) maintained its structure to grow at the first stage, and they did not transform into MOF(l) during the growth process.

Thus, following the fast growth around the nucleation sites, the MOF(l) layer gradually grow on the (001) facet of the primary MOF(s) (Fig. 4d and Supplementary Fig.35) at 2h. Furthermore, it reached the silmilar lateral size and orientation with the primary layer at 3 h, confirmed by both the top view (Fig. 4e ans Supplementary Fig. 23.) and side view (Fig.4g), and the corresponding TEM images of the two facet are shown in Fig. 4f,h respectively. It was foundt that two sets of SAED diffraction spots already emerged (Fig. 4e-h and Supplementary Fig. 36).

Considering the more thermodynamically stable crystal structure of MOF(l), the growth speed of MOF(l) should be faster than MOF(s). Subsequently, both layers continued to grow, mainly in two dimensions due to the attachment of PVP in (100) surface. For the 6 h sample, one nanolayer was already slightly larger than the other (Fig. 4i,k in the main text and Supplementary Fig. 37). Consistently, two sets of (100) diffraction spots were observed (inset in Fig. 4j,l). And the smaller layer was partially embedded into the large layer, likely resulting from different growth rates along the [001] axis of both nanoplates. As a result, Co-MOF-3 nanostacks with slightly different crystal phases were prepared. The schematic diagram of the formation mechanism was summarized in Fig. 4m.

It may be argued that the formation of both nanoplates and nanostacks is somewhat related to the well-known "oriented attachment" of smaller pieces. However, since we did not observe at any time non-perfectly aligned nanoplates, we excluded this possibility. Another strong proof for the proposed mechanism of formation came from absorption measurements. The reaction solution's UV-vis spectra show a gradual decrease of the peak of TCPP at 415 nm with reaction time, indicating that TCPP molecules were gradually consumed to feed the synthesis of Co-MOF until at least 20 h (Supplementary Fig. 38). Combining all these results, we hypothesize that the anisotropic growth of Co-MOF-3 nanostacks is induced by the Au/Ag nanocubes, thanks to the weak coordination interaction between -COOH and Au/Ag. As shown in Fig. 4m, the Au/Ag nanocubes direct the initial heterogeneous nucleation of the first Co-MOF nanolayer, followed by the homogeneous nucleation of the second Co-MOFs nanolayer due to the similar composition and the same interplanar distance in the vertical axis. The smaller layer was partially embedded into the large layer, likely resulting from different growth rates along the [001] axis of both nanoplates.

## 3. Supplementary Figures

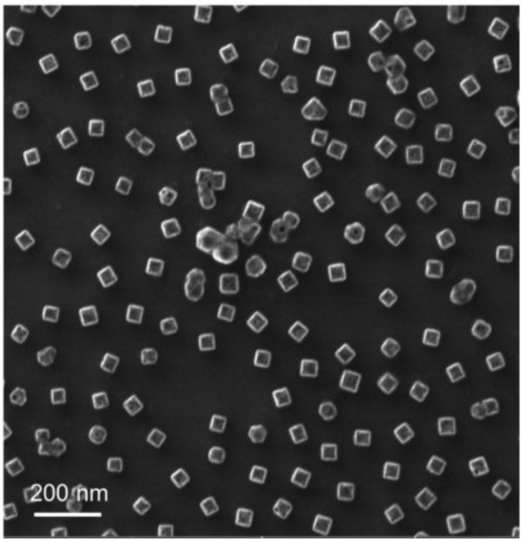


**Supplementary Figure 1.** Typical SEM image of hollow Ag/Au nanocubes with Ag/Au ratio of 47:53.

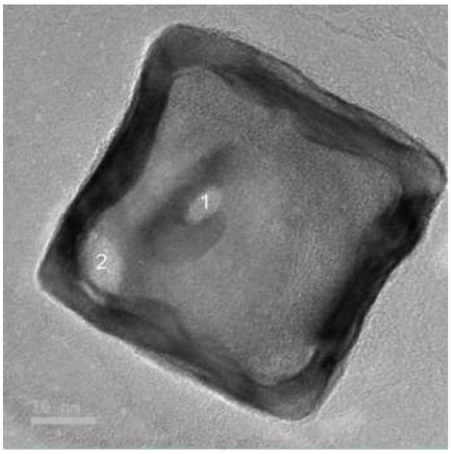


**Supplementary Figure 2.** A typical TEM image of a hollow Ag/Au nanocube with Ag/Au ratio of 47:53 (the white part of 1 and 2 represent the pores in the nanocube, suggesting its non-fully compact structure).

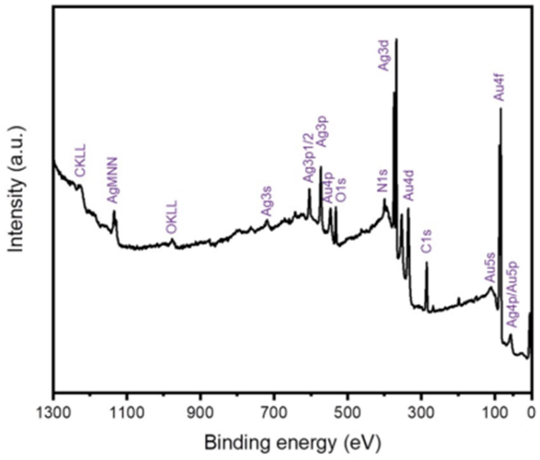

**Supplementary Figure 3.** XPS spectrum of hollow Ag/Au nanocubes with Ag/Au ratio of 47:53.

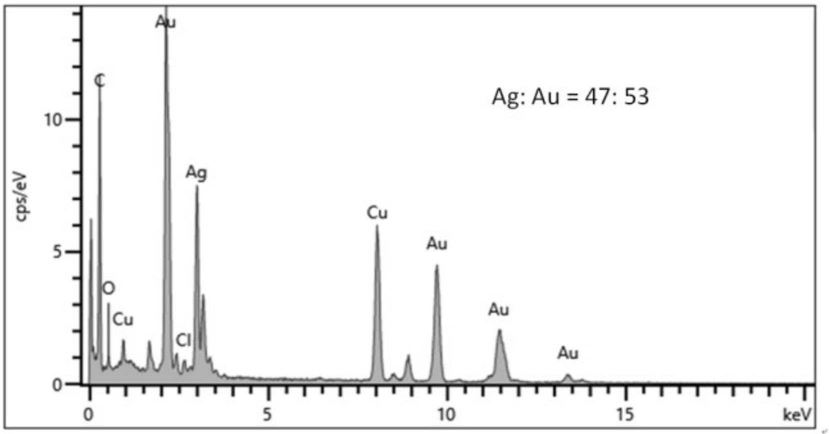


**Supplementary Figure 4.** EDS spectrum of hollow Ag/Au nanocubes used to induce formation of Co-MOF-3.

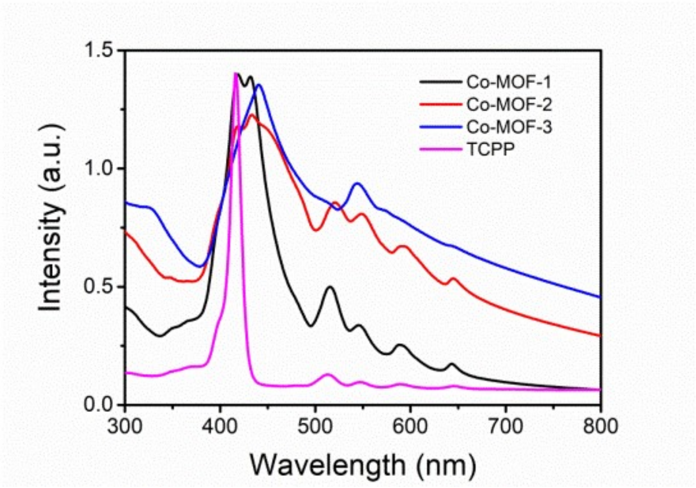


**Supplementary Figure 5.** UV-vis absorption spectra of Co-MOF-1, Co-MOF-2, Co-MOF-3 and TCPP in ethanol solution.

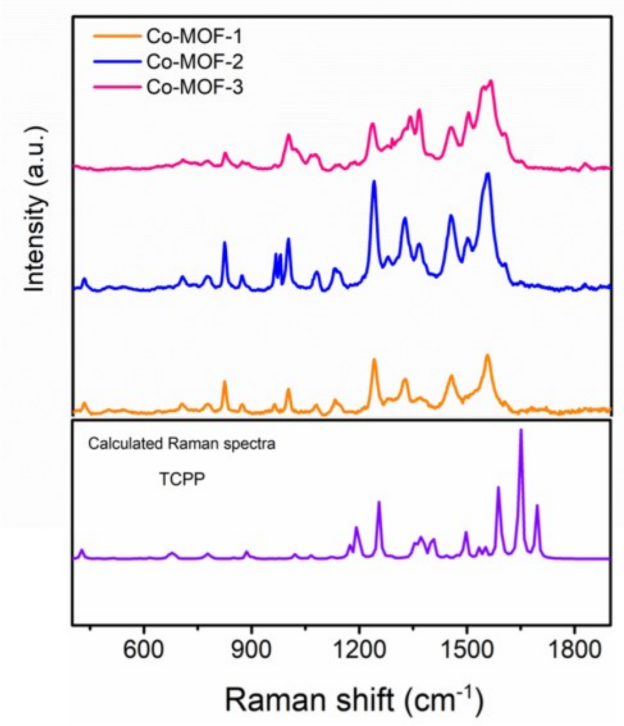


**Supplementary Figure 6.** Raman spectra of Co-MOF-1, Co-MOF-2, Co-MOF-3 and TCPP.

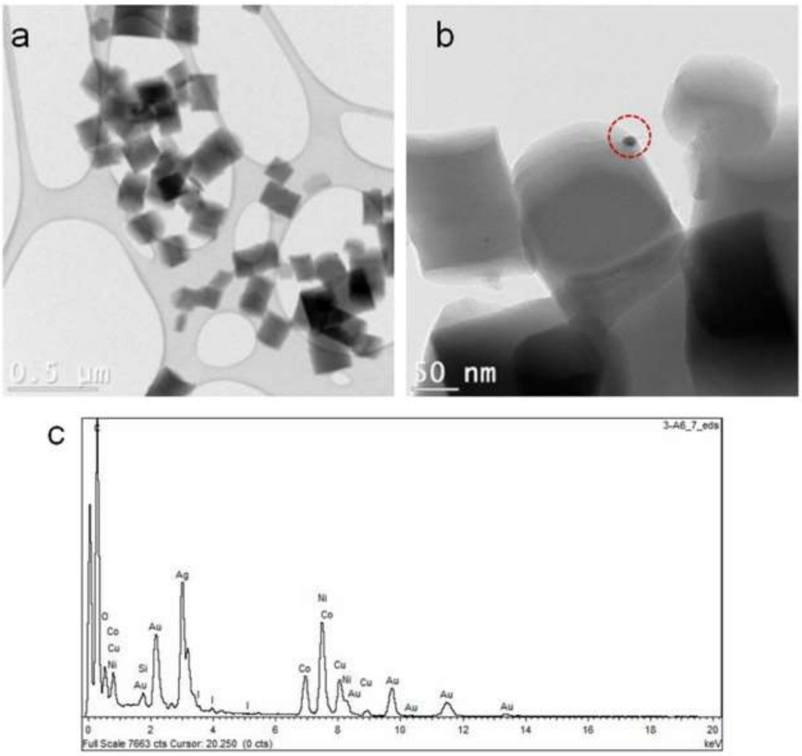


**Supplementary Figure 7.** (a, b) Representative TEM images of Co-MOF-1. (c) EDS spectrum of the small dark nanoparticle highlighted by the red cycle in (b) (Ni comes from the Cu grid). A few small "dark" spherical NPs with a diameter smaller than 15 nm, marked by red cycles, were observed in some MOFs (b), but no hollow nanocubes could be found in the TEM images. EDS analysis showed that these spherical NPs contained Ag and Au

elements (c), which implies that the hollow Au/Ag nanocubes have been consumed during the synthesis process of MOFs and transformed to smaller spherical NPs.

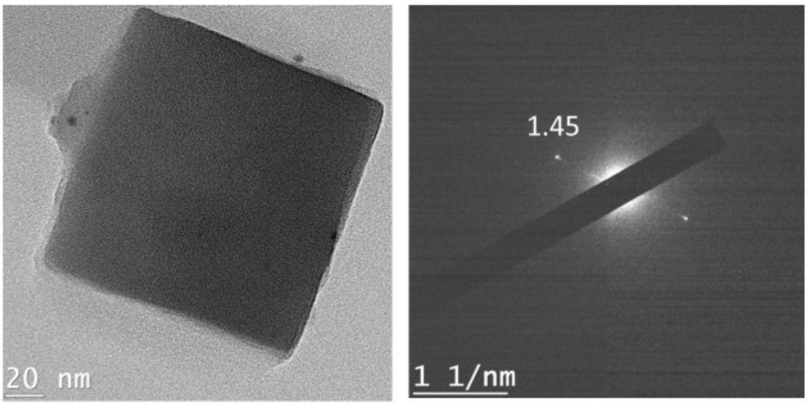


**Supplementary Figure 8.** (a) Representative HR-TEM images of Co-MOF-1 and the corresponding SAED pattern.

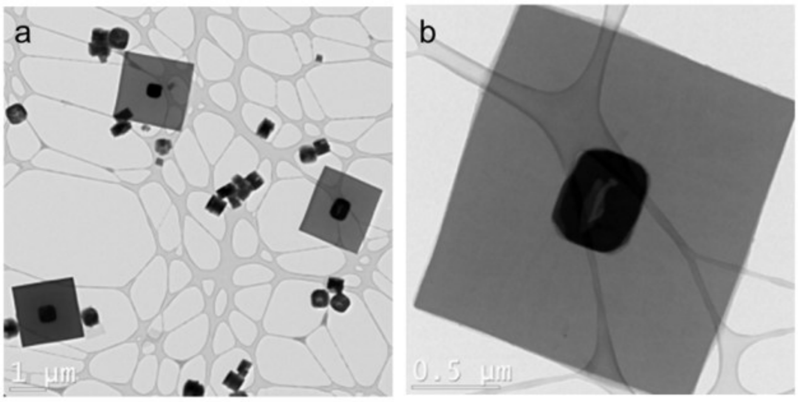


**Supplementary Figure 9. (a)** Representative TEM image of Co-MOF-2. (b) Magnified TEM image of stacked nanoplates in Co-MOF-2.

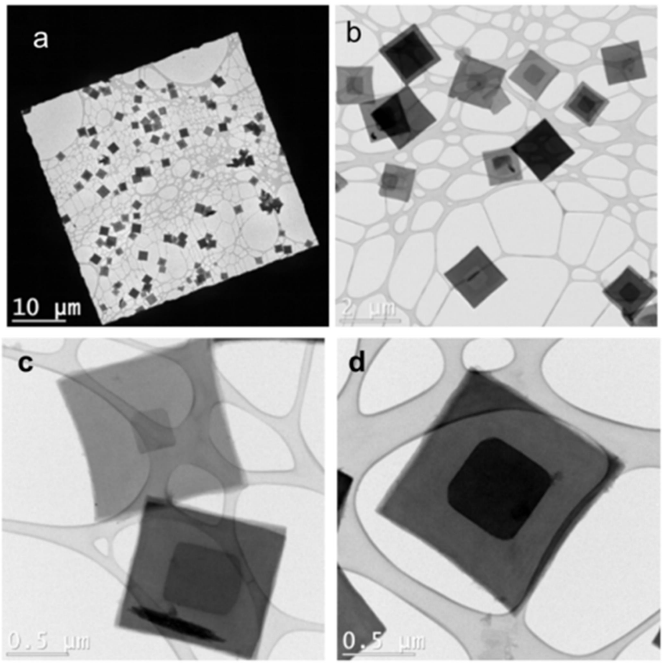


**Supplementary Figure 10.** Representative TEM images of Co-MOF-3 nanostacks with different magnifications.

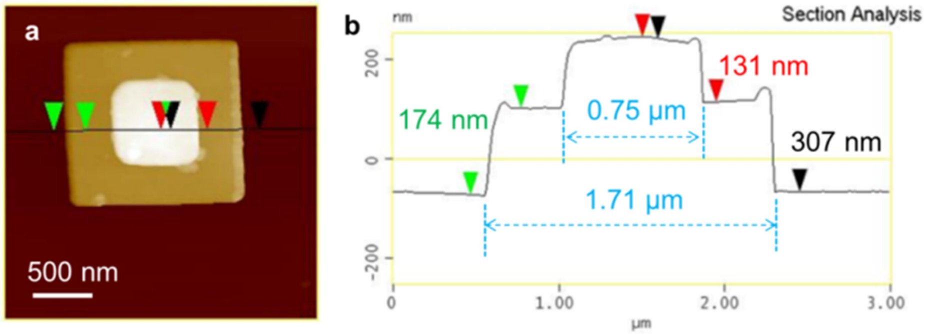


**Supplementary Figure 11.** AFM image of Co-MOF-3 (a) and the corresponding height profile (b). It showed the height (vertical distance) and edge length (horizontal distance) of the large nanoplate were 174 nm and 1.71 μm, respectively. In contrast, the height and edge length of the smaller nanoplate were 131 nm and 0.75 μm, respectively.

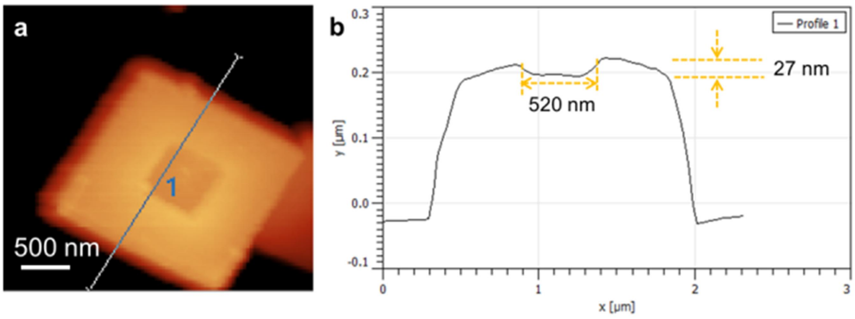

**Supplementary Figure 12.** AFM image of Co-MOF-3 showing a pit in the center (a) and the corresponding height profile (b).

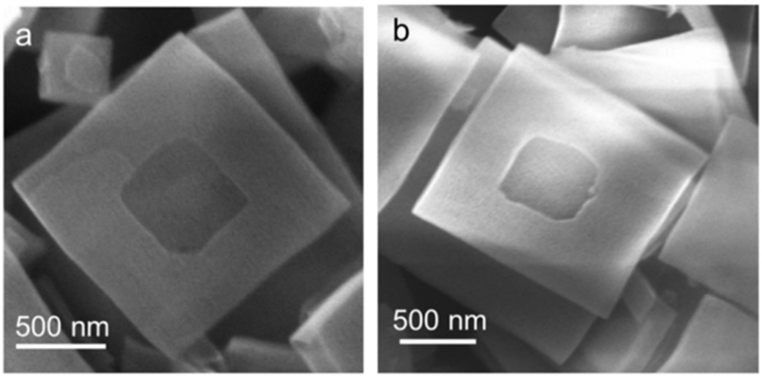


**Supplementary Figure 13.** SEM images of Co-MOF-3 showing a pit in the center.

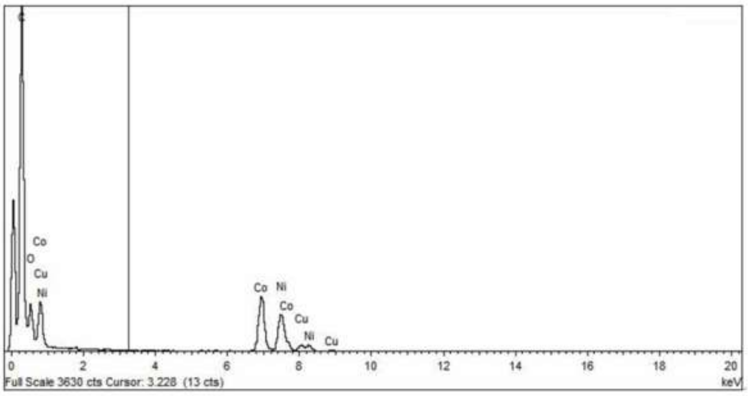


**Supplementary Figure 14.** EDS spectrum of the Co-MOF-3 (Ni comes from the Ni grid).

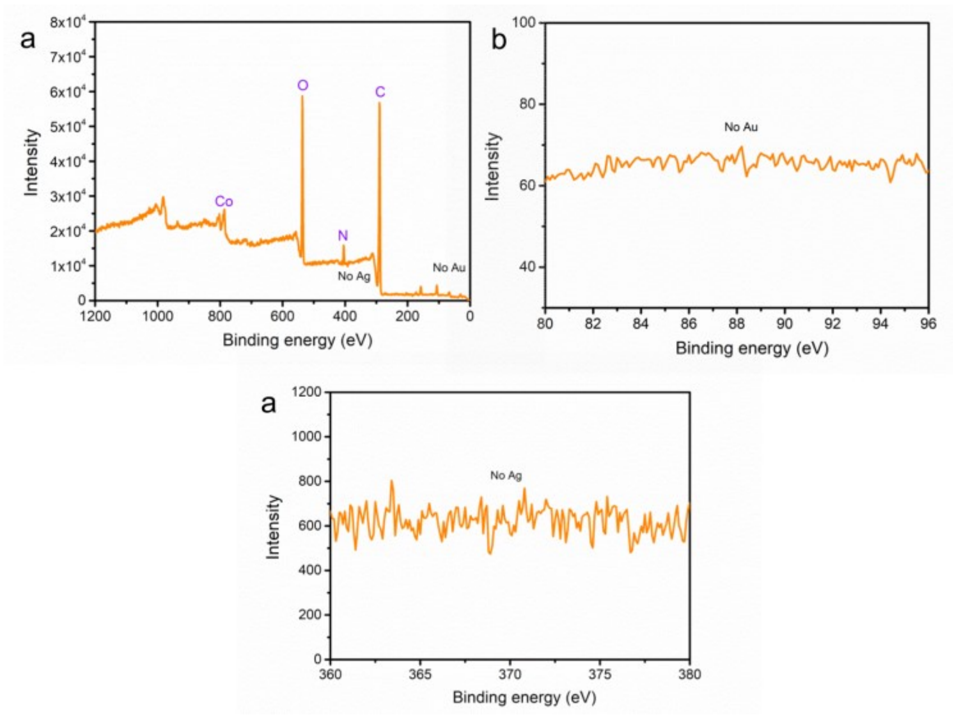


**Supplementary Figure 15.** XPS spectra of Co-MOF-3 with (a) full range, (b) 80 eV - 96 eV and (c) 360- 380 eV, respectively, suggesting the presence of Co element, but no apparent Au and Ag elements left in the samples.

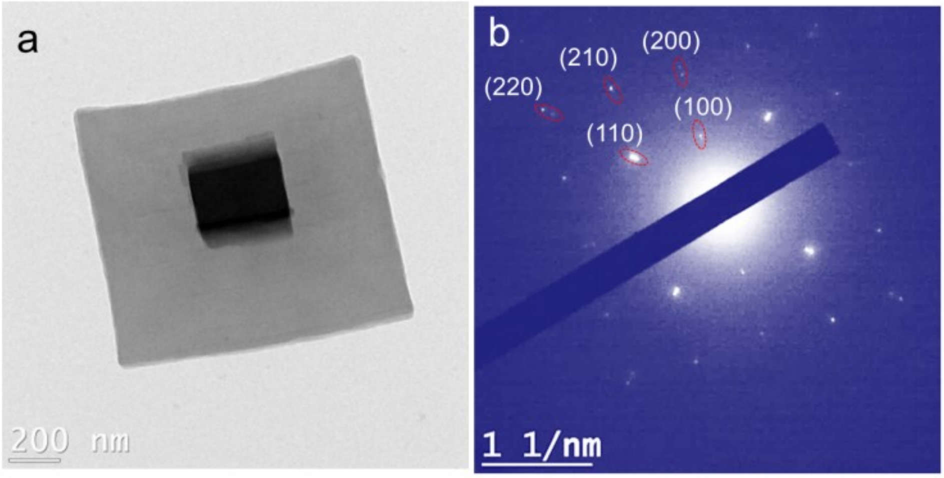


**Supplementary Figure 16.** (a) TEM image and (b) the corresponding SAED of Co-MOF-3 stacked nanoplates showing multi-order diffraction spot of (100), (110), (200), (210) and (220) facet.

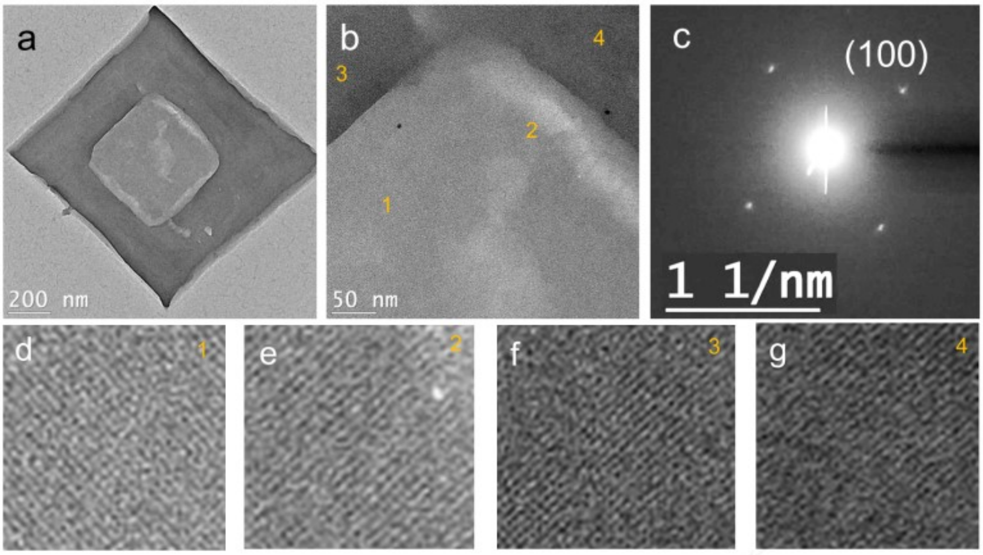


**Supplementary Figure 17.** TEM images of a Co-MOF-3 nanoplate showing a pit in the center (a,b) and the corresponding FFT image (c). (d) (e) (f) (g) enlarged-TEM images from (b) at position 1, 2, 3, 4, respectively.

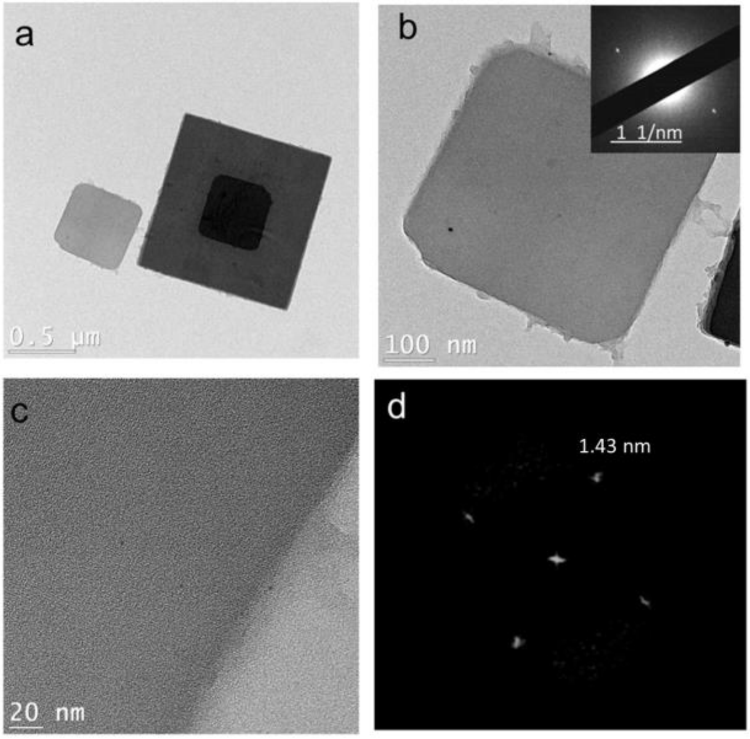


**Supplementary Figure 18.** TEM images of Co-MOF-3 nanoplates (a) and one MOF(s) nanoplate (b), (c) HR-TEM of MOF(s) and (d) the corresponding FFT image in (c).

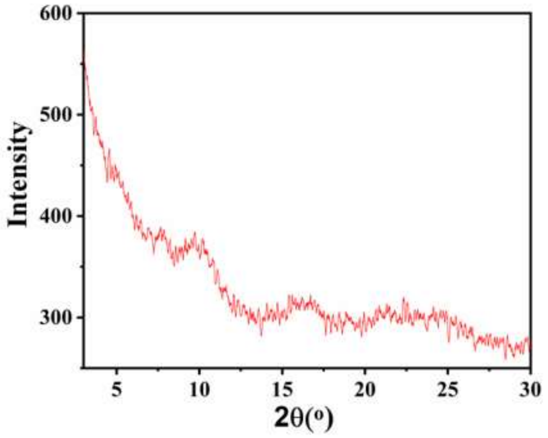


**Supplementary Figure 19. XRD pattern of Co-MOF-3.**

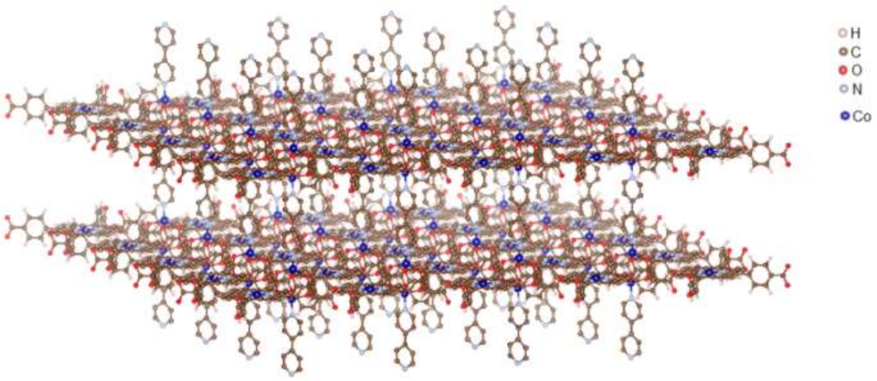


**Supplementary Figure 20.** 3D crystal structural model of MOF(c) with AA stacking.

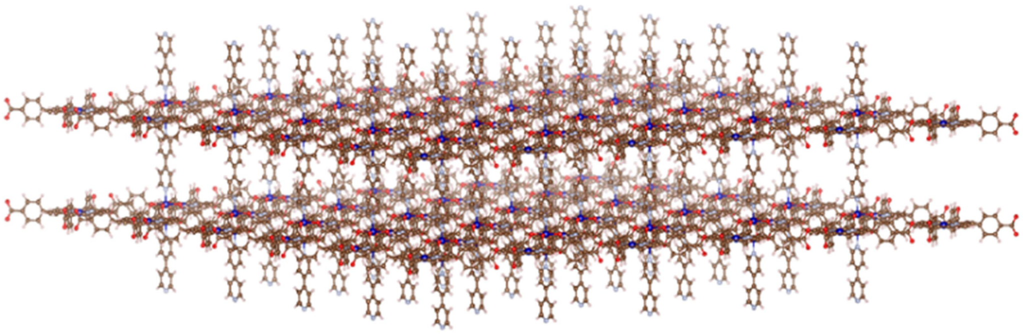

**Supplementary Figure 21.** 3D crystal structural model of MOF(e) with AA stacking.

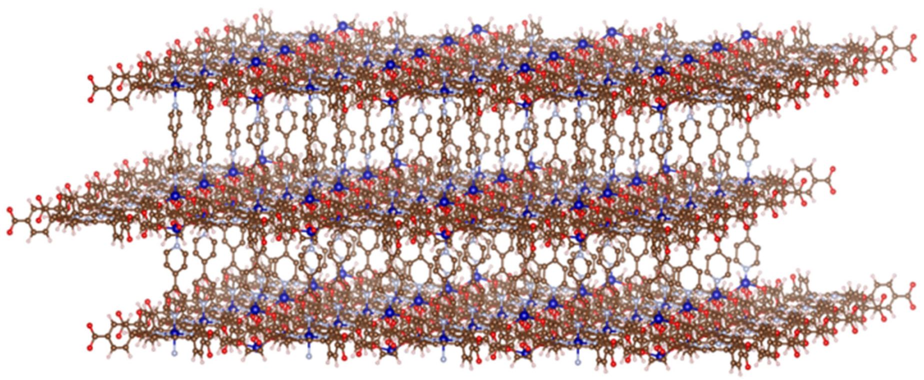

**Supplementary Figure 22.** 3D crystal structural model of MOF(c) in a form of AB stacking.

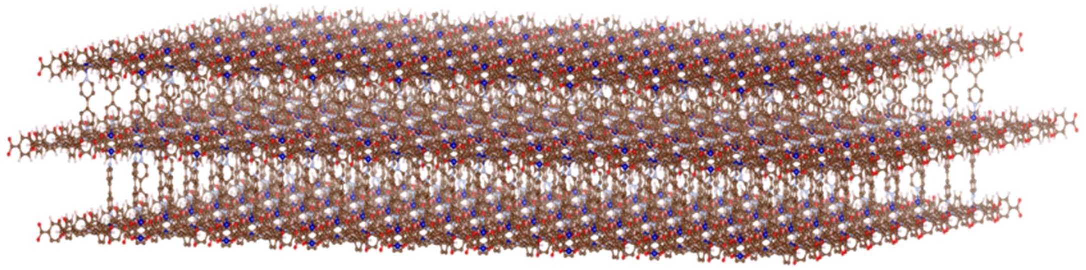

**Supplementary Figure 23.** 3D crystal structural model of MOF(c) in a form of AB stacking.

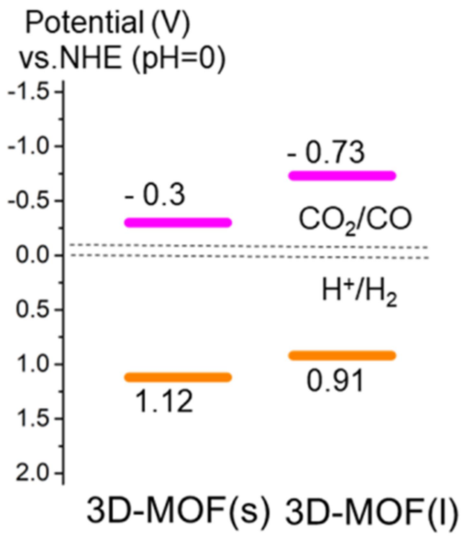


**Supplementary Figure 24.** Calculated energy levels of 3D MOF(s) and MOF(l) with AA stacking (Potential vs. NHE (pH = 0)).

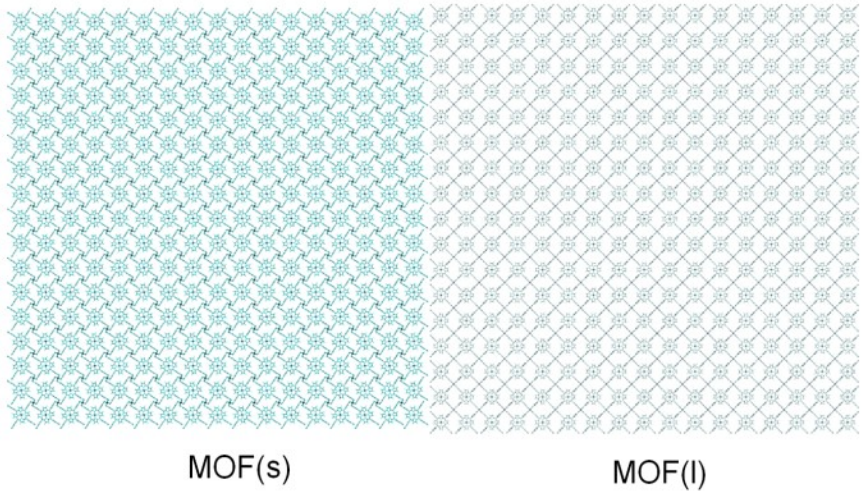


**Supplementary Figure 25.** Schematic illustrations of side contact between MOF(s) and MOF(l) in Co-MOF-3.

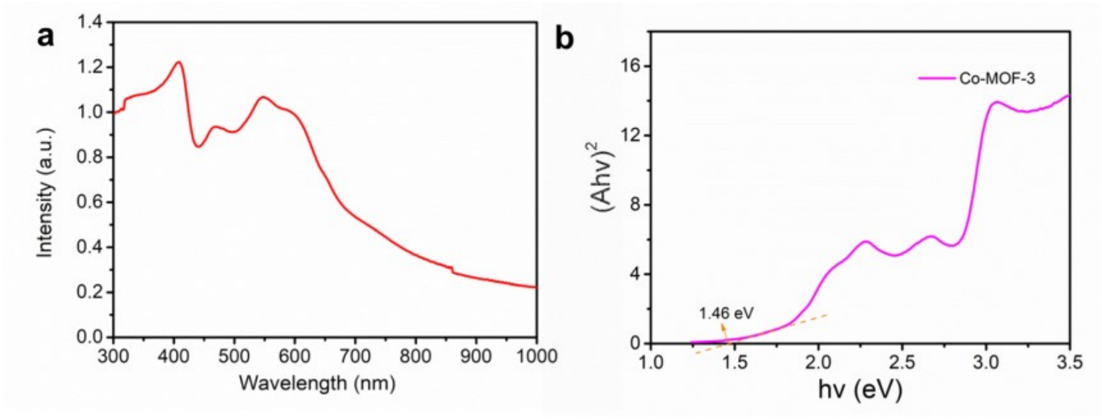


**Supplementary Figure 26.** (a) UV–Vis diffuse reflectance spectra and (b) Tauc plot of the Co-MOF-3.

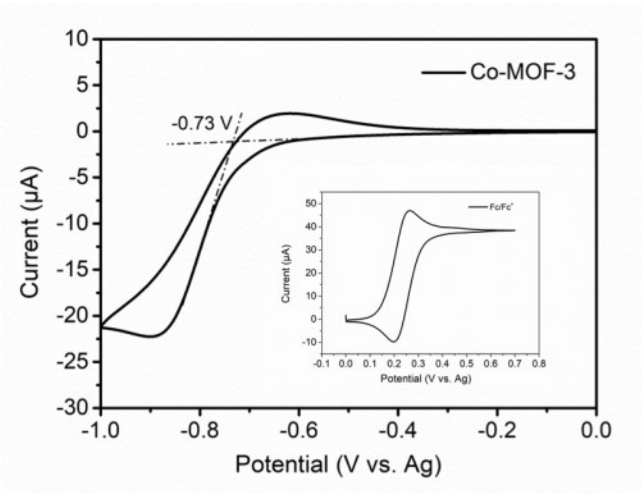


**Supplementary Figure 27.** CV curves of Co-MOF-3 at a scan rate of 10 mV $s^{-1}$ (inset: CV of ferrocenium/ferrocene).

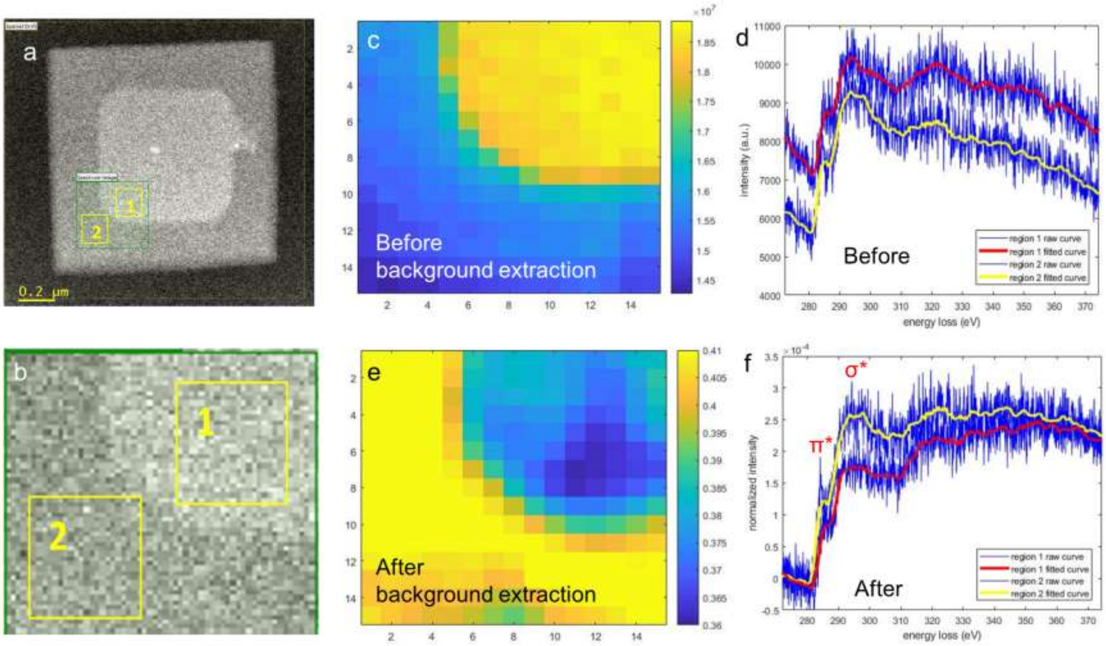


**Supplementary Figure 28.** (a) HADDF image of one Co-MOF nanostack and (b) enlarged image of selected area in Co-MOF, Raw data of EELS mapping (c) and spectra (d) in the carbon K edges region acquired from different regions of Co-MOF nanostack. EELS mapping (e) and spectra (f) extracted with background removal and smoothed by Gaussian fitting method.

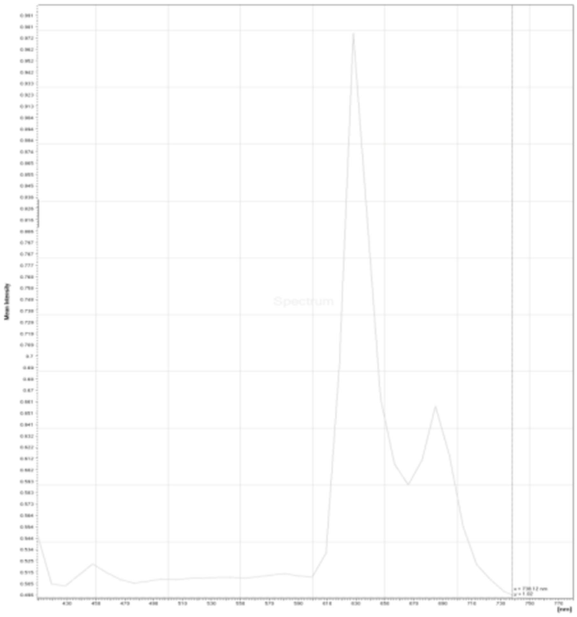

**Supplementary Figure 29.** FL spectrum of one Co-MOF-3 from super-resolution multi-photon confocal microscope.

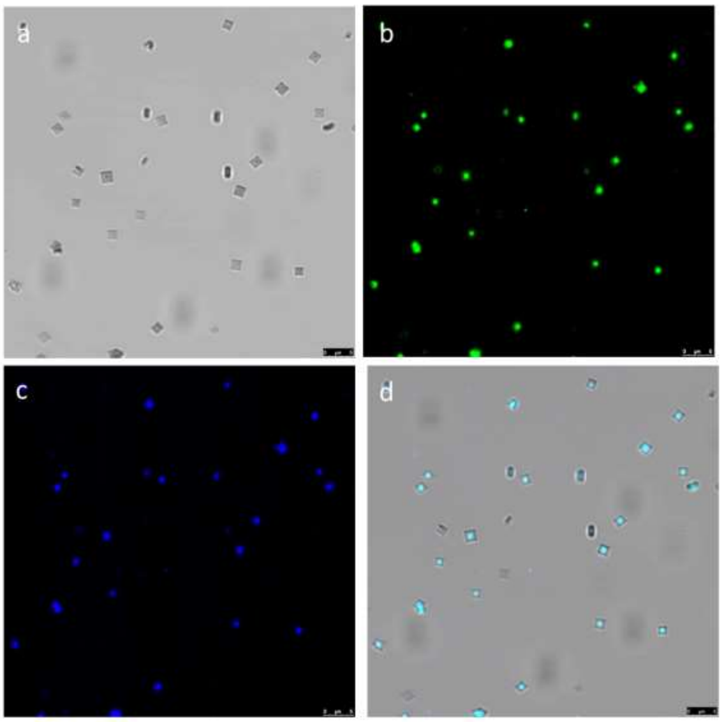


**Supplementary Figure 30.** Super-resolution Multiphoton Confocal images of Co-MOF-3: (a) bright field, (b,c) FL, and (d) merge images. The excitation of wavelength is 405 nm.

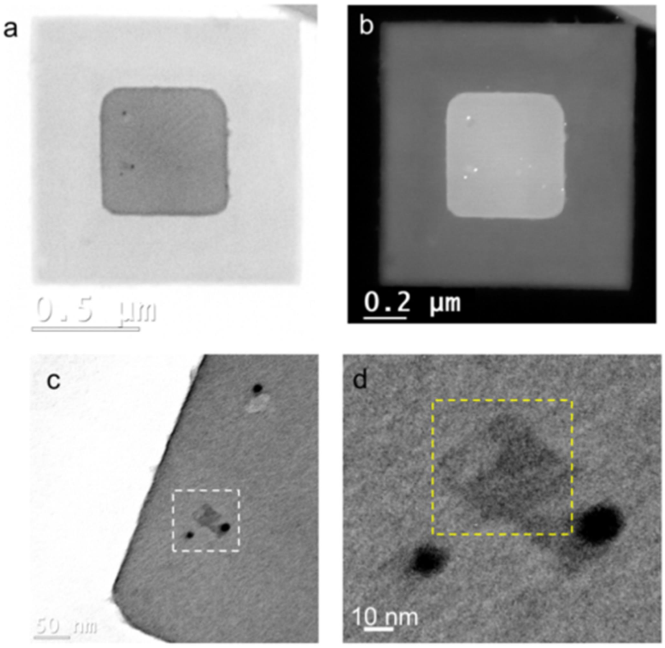


**Supplementary Figure 31.** TEM image (a) and HAADF images (b) of Co-MOF-3 with Ag/Au nanoparticles.(c, d) Enlarged TEM images on small nanoplate (the yellow square may indicate the inducer of a Ag/Au nanocube, the outline of which is discernible).

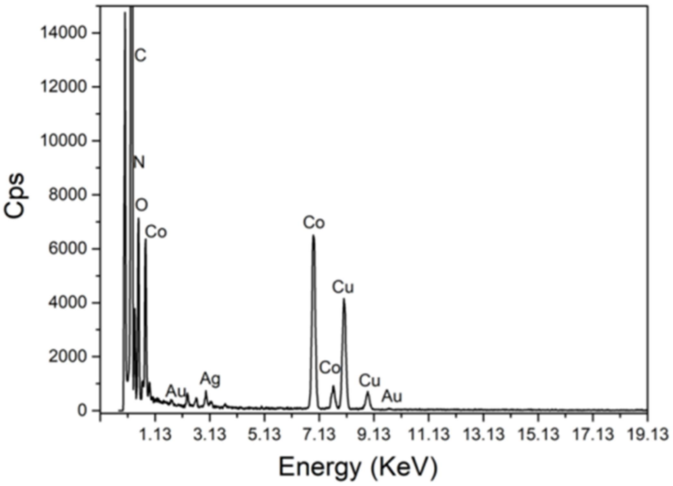


**Supplementary Figure 32.** EDS of Ag/Au NPs in Co-MOF-3 (Cu signal is from the Cu grid)**.**

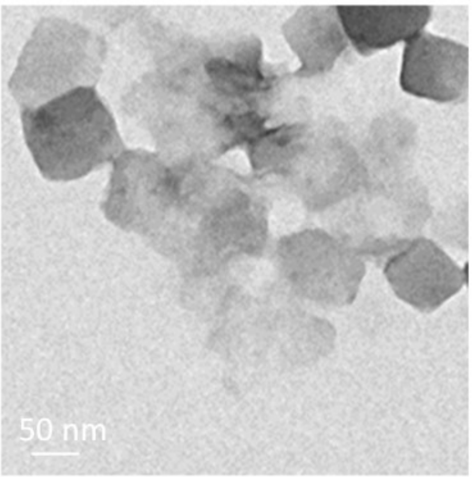


**Supplementary Figure 33.** TEM images of Co-MOF-3 at 15 min reaction time.

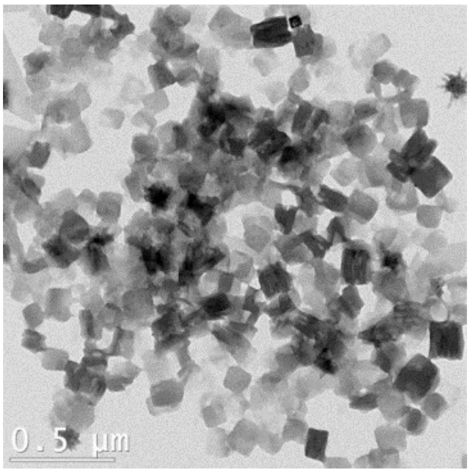


**Supplementary Figure 34.** TEM images of Co-MOF-3 at 1h reaction time.

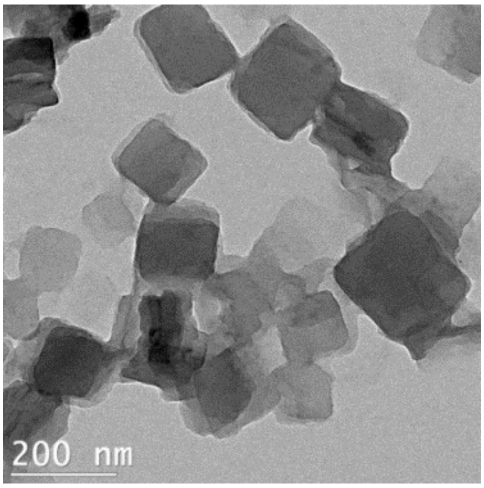


**Supplementary Figure 35.** TEM images of Co-MOF-3 at 2h reaction time.

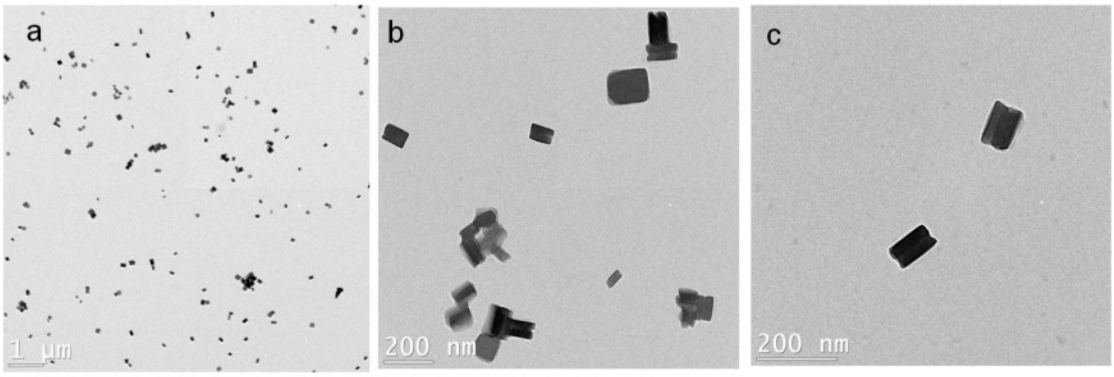


**Supplementary Figure 36.** TEM images of Co-MOF-3 at 3h reaction time.

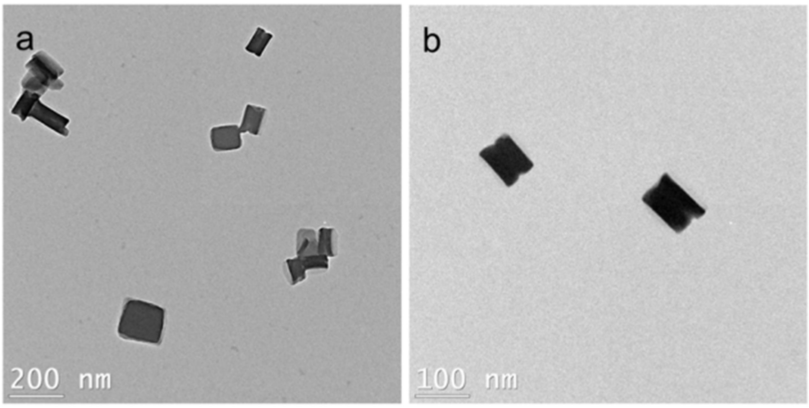


**Supplementary Figure 37.** TEM images of Co-MOF-3 at 6h reaction time.

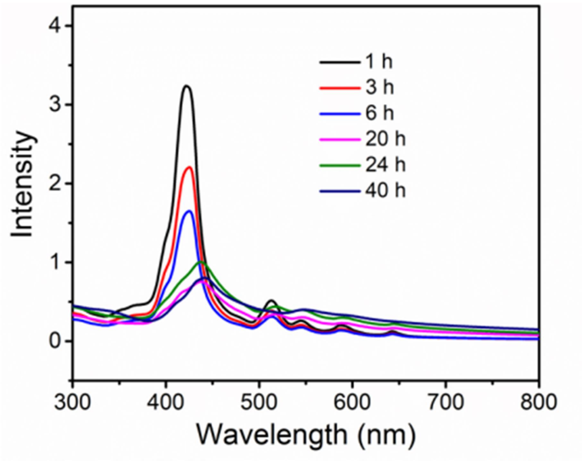


**Supplementary Figure 38.** UV-vis spectra of Co-MOF-3 in ethanol solution taken at various reaction time.

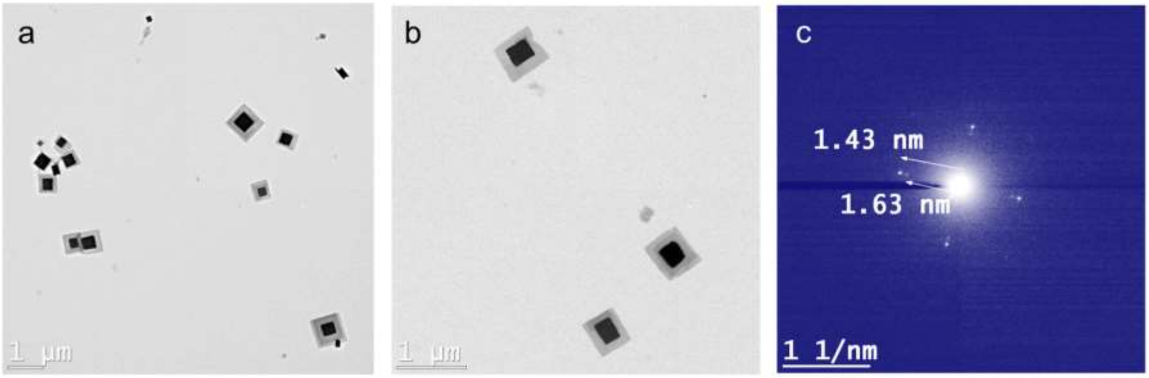


**Supplementary Figure 39.** (a,b) TEM images of Co-MOF nanoplates with the amount of Au/Ag cube of 15 μ mol and the corresponding SAED image.

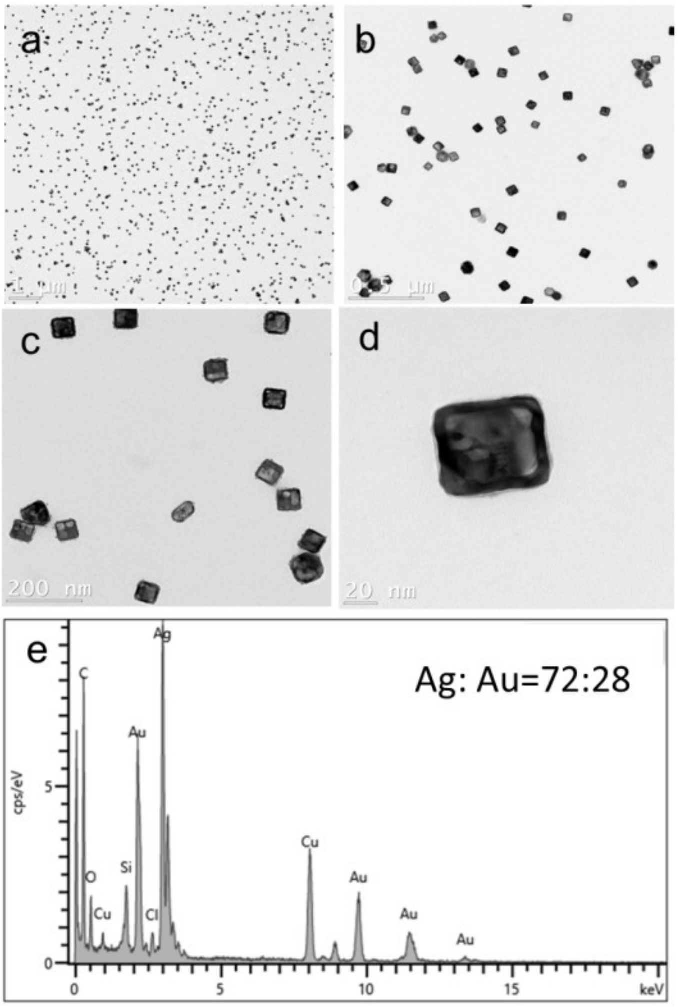


**Supplementary Figure 40.** Typical TEM images (a-d) of the hollow Au/Ag nanocubes and the corresponding EDS spectrum with Ag/Au ratio of 72:28.

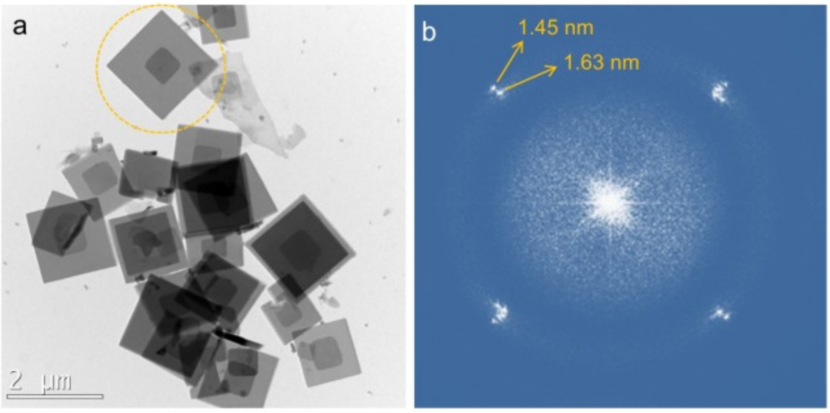


**Supplementary Figure 41.** (a) TEM images of Co-MOF nanoplates induced by hollow Au/Ag cube with Ag/Au ratio of 72:28 and (b) the corresponding SAED image.

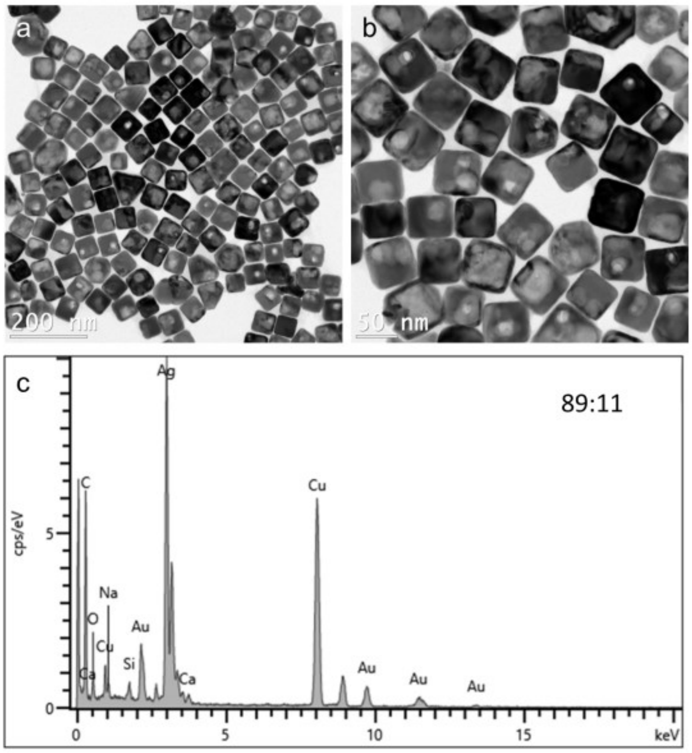


**Supplementary Figure 42.** Typical TEM images (a-d) of the hollow Au/Ag nanocubes and the corresponding EDS spectrum with Ag/Au ratio of 89:11.

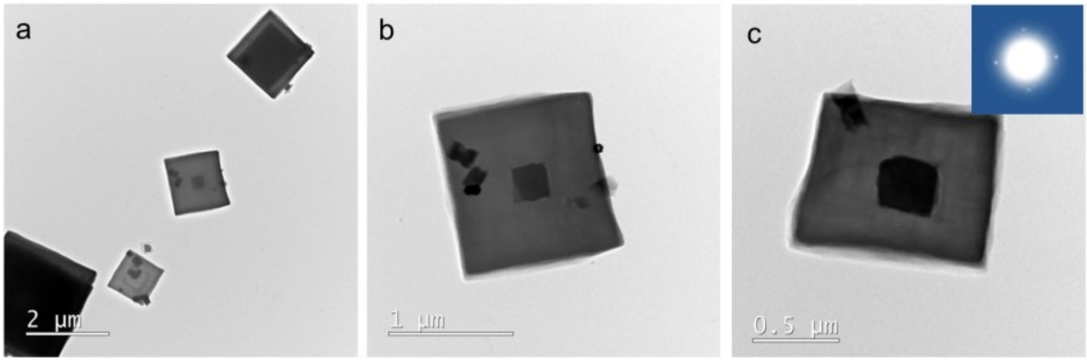


**Supplementary Figure 43.** (a-c) TEM images of Co-MOF nanostacks induced by hollow Au/Ag cube with Ag/Au ratio of 89:11 and the corresponding SAED image (inset in c).

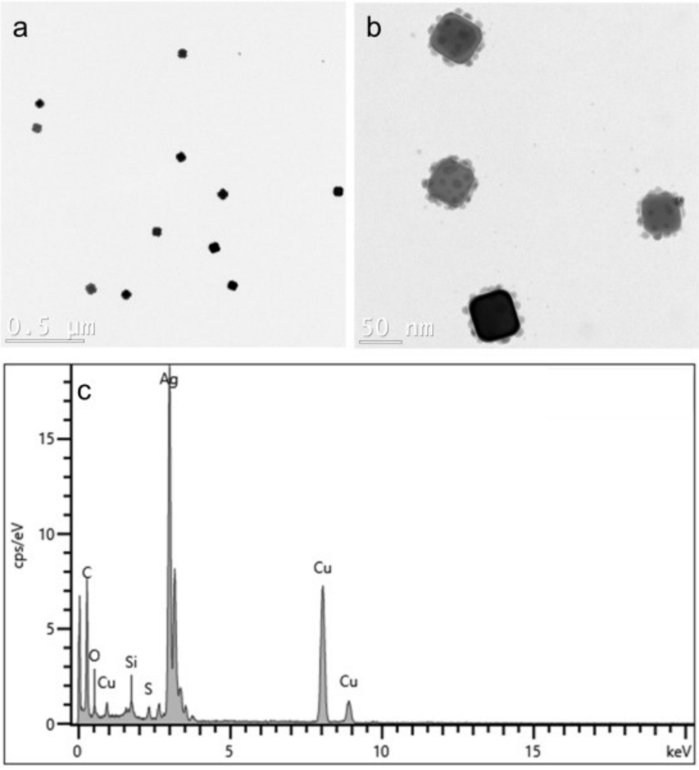


**Supplementary Figure 44.** Typical TEM images (a-d) of the Ag nanocubes and the corresponding EDS spectrum.

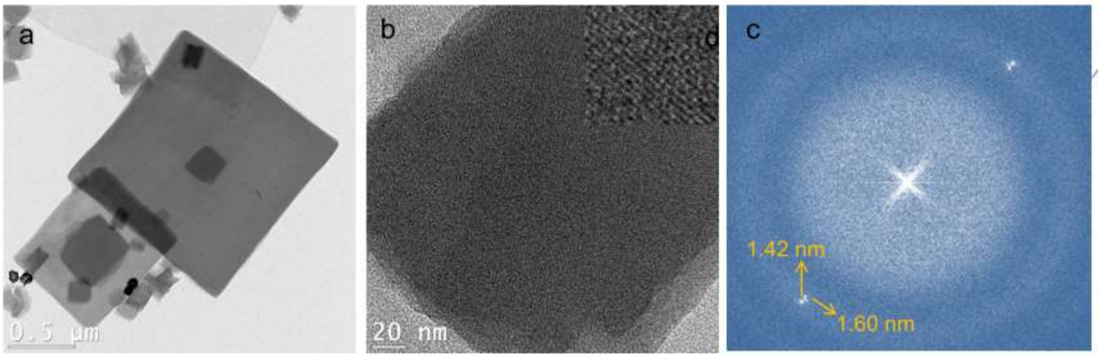


**Supplementary Figure 45.** Typical TEM images (a-d) of the Ag nanocubes and the corresponding EDS spectrum.

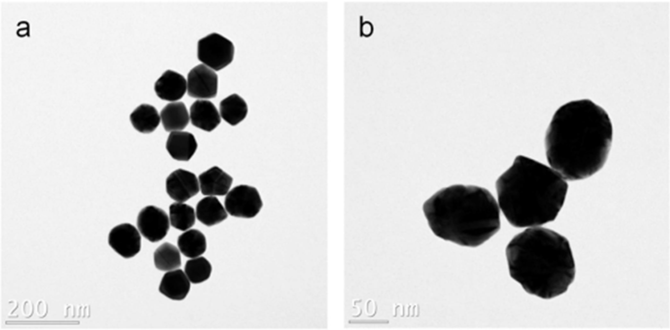


**Supplementary Figure 46.** Typical TEM images of the Au NPs.

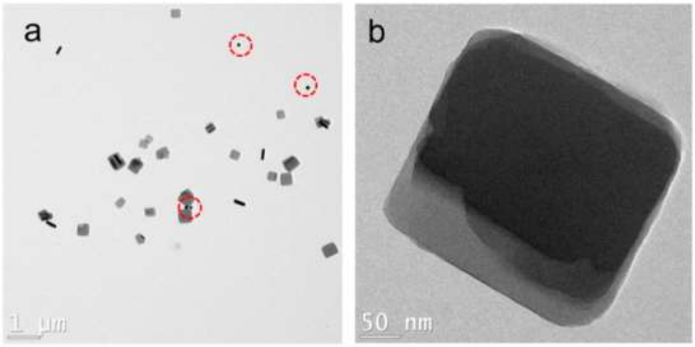


**Supplementary Figure 47.** TEM images of Co-MOF at 3 h reaction time induced by Au nanoparticles and red circles highlight AuNPs.

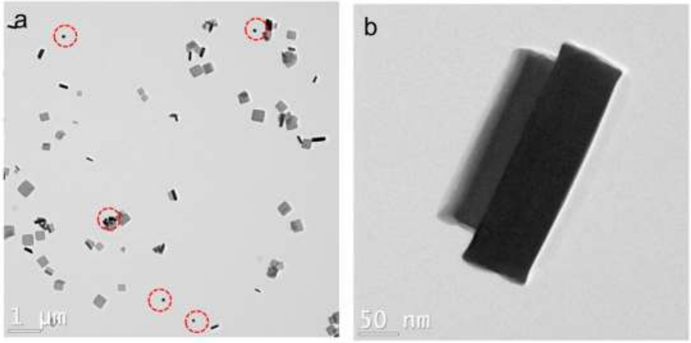


**Supplementary Figure 48.** TEM images of Co-MOF at 6 h reaction time induced by Au nanoparticles and red circles highlight AuNPs.

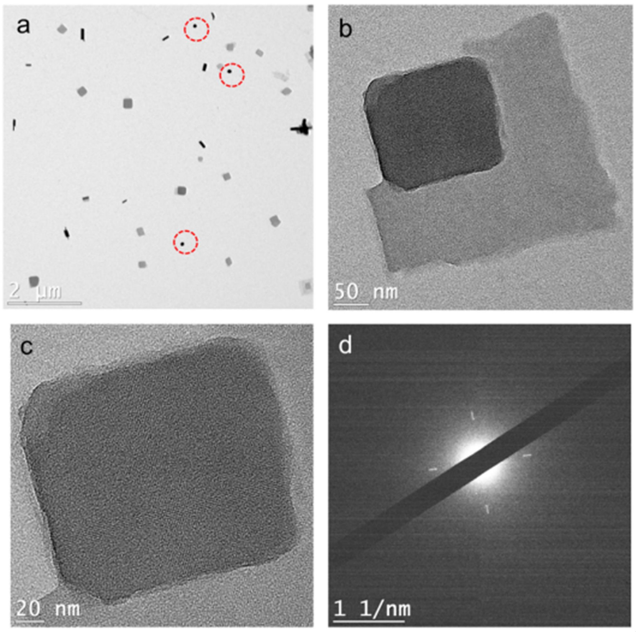


**Supplementary Figure 49.** (a,b) TEM images of Co-MOF at 24 h reaction time induced by Au NPs, and red circles highlight AuNPs. (c) HR-TEM image of Co-MOF and (d) corresponding SAED image.

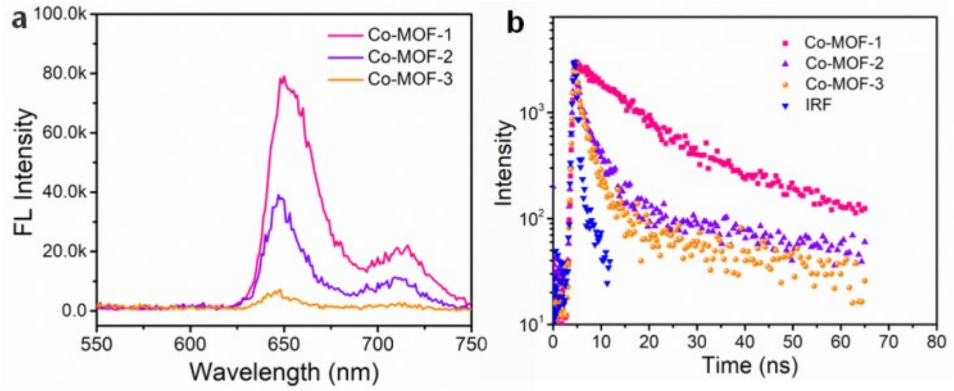


**Supplementary Figure 50.** Photogenerated charge separation mechanism of Co-MOF-3 homojunction. (a) The FL spectra of the Co-MOF-1, Co-MOF-2 and Co-MOF-3 with the same concentration in ethanol solution (Excitation:430 nm). (b) The FL decay traces of the Co-MOF-1, Co-MOF-2 and Co-MOF-3 (Excitation:430 nm and Emission: 650 nm).

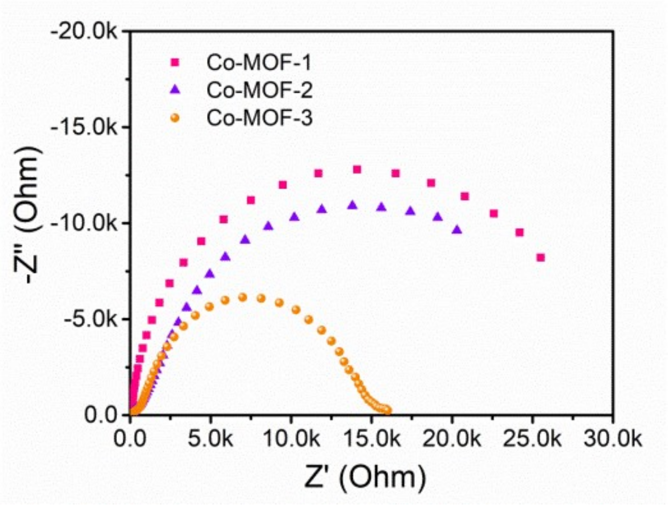


**Supplementary Figure 51.** Nyquist plots for the Co-MOF-1, Co-MOF-2 and Co-MOF-3 at a bias of 0 V *vs.* RHE from electrochemical impedance spectra (EIS) tests.

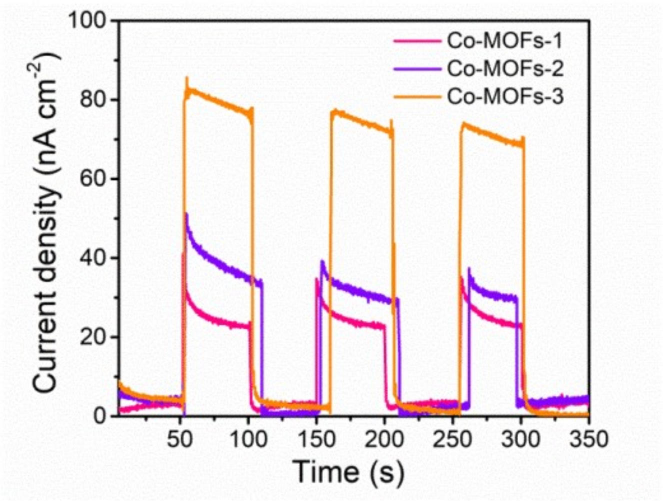


**Supplementary Figure 52.** Photocurrent-time plots of the Co-MOF-1, Co-MOF-2 and Co-MOF-3 at a bias of 0.2 V under AM 1.5G irradiation and dark conditions (near-zero regions).

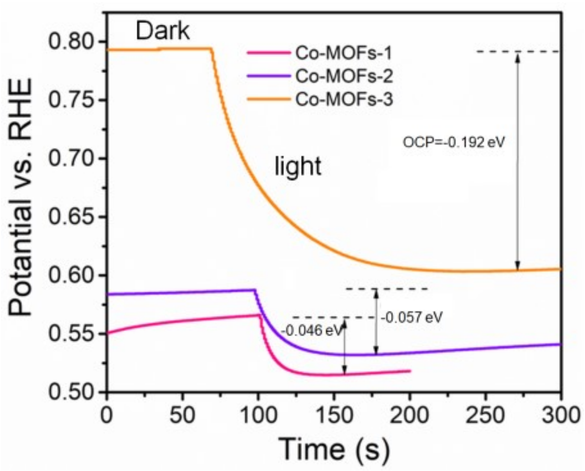


**Supplementary Figure 53.** OCP response curves of Co-MOF-1, Co-MOF-2 and Co-MOF-3 under dark or AM 1.5G irradiation conditions.

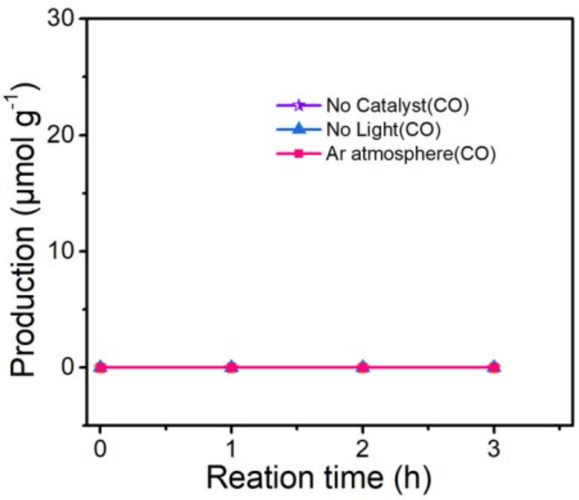


**Supplementary Figure 54.** Evolution of CO and $H_2$ as a function of reaction time in aqueous solution under visible light illumination for the photocatalysts (No catalyst: only no Co-MOF-3 in system; No light: no illumination; Ar atmosphere: $CO_2$ was changed to be Ar with other same condition).

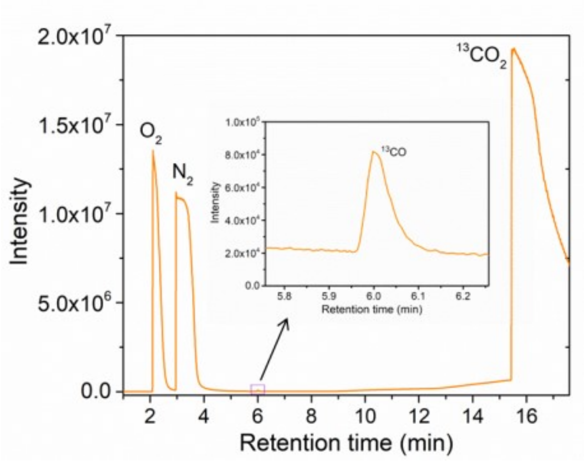


**Supplementary Figure 55.** Total gas chromatography spectra after the visible light reduction of $^{13}CO_2$ in water solution using Co-MOF-3 as a photocatalyst.

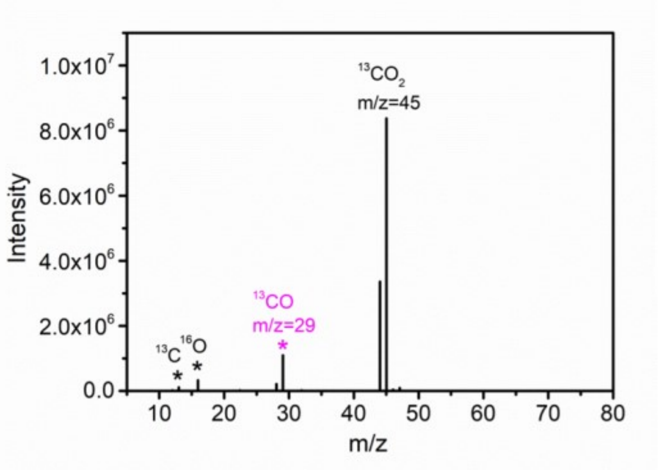


**Supplementary Figure 56.** Mass spectrum of $^{13}CO_2$ using Co-MOF-3 as a photocatalyst.

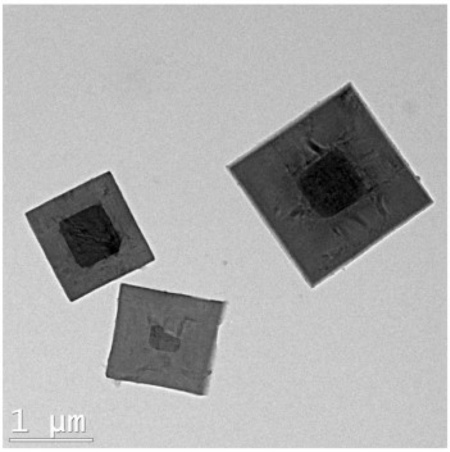


**Supplementary Figure 57.** Morphology of Co-MOF-3 after photocatalyst.

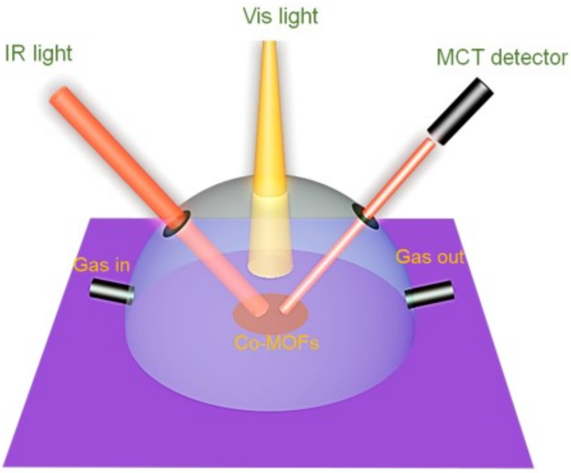


**Supplementary Figure 58.** In situ diffuse reflectance FT-IR (DRIFTS) setup for monitoring the $CO_2$ photoreduction mechanism.

**Supplementary Table 1.** Ratios of K series peak area of Co/N, Co/O and Co/C in three different Co-MOF-3 stacked nanoplates.

| **Co-MOF-3 nanostacks-1** | Co/N | Co/O | Co/C |
|---|---|---|---|
| EDS2 (center part) | **4.19** | **1.86** | **0.14** |
| EDS1 (edge part) | **3.76** | **1.81** | **0.13** |
| EDS2/EDS1 | **1.11** | **1.03** | **1.08** |
| **Co-MOF-3 nanostacks-2** | | | |
| EDS2 (center part) | **4.35** | **1.87** | **0.21** |
| EDS1 (edge part) | **3.52** | **1.62** | **0.19** |
| EDS2/EDS1 | **1.23** | **1.15** | **1.10** |
| **Co-MOF-3 nanostacks-3** | | | |
| EDS2 (center part) | **5.49** | **2.76** | **0.21** |
| EDS1 (edge part) | **4.68** | **2.46** | **0.19** |
| EDS2/EDS1 | **1.17** | **1.12** | **1.10** |

**Supplementary Table 2.** The Co-MOF nanostacks under different inducer agents of Ag/Au nanocubes with varied Ag/Ag ratios, AgNPs and AuNPs (Element ratios obtained from EDS).

| Inducer | Co-MOF Nanostacks | Sets of SAED patterns |
|---|---|---|
| Hollow Ag/Au nanocube (Ag/Au=47:53) | Yes (91%) | Two sets |
| Hollow Ag/Au nanocube (Ag/Au=72:28) | Yes | Two sets |
| Hollow Ag/Au nanocube (Ag/Au=89:11) | Yes | One set |
| AuNPs | Yes | Two sets |

| | | |
|---|---|---|
| AgNPs | Yes | Two sets |

## 4. Supplementary References


1. Mielke, J. *et al.* Adatoms underneath single porphyrin molecules on Au(111). *J. Am. Chem. Soc.* **137**, 1844-1849 (2015).
2. Krasnikov, S.A., Doyle, C.M., Sergeeva, N.N. *et al.* Formation of extended covalently bonded Ni porphyrin networks on the Au(111) surface. *Nano Res.* **4**, 376-384 (2011).
3. Kudernac, T., Lei, S., Elemans, J. A. A. W. & De Feyter, S. Two-dimensional supramolecular self-assembly: nanoporous networks on surfaces. *Chem. Soc. Rev.* **38**, 402-421(2009).
4. Yoshimoto, S. Effects of protonation of pyridine moieties on the 2D assembly of porphyrin layers on Au(111) at electrochemical interfaces. *Chem. Commun.* **48**, 4612-4614 (2012).
5. Blunt, M. O. *et al.* Controlling the two-dimensional self-assembly of functionalized porphyrins via adenine–thymine quartet formation. *J. Phys. Chem. C* **122**, 26070-26079 (2018).
6. El Garah, M. *et al.* Molecular design driving tetraporphyrin self-assembly on graphite: a joint STM, electrochemical and computational study. *Nanoscale* **8**, 13678-13686 (2016).
7. Slater, A. G. *et al.* Thymine functionalized porphyrins, synthesis and heteromolecular surface-based self-assembly. *Chem. Sci.* **6**, 1562-1569 (2015).
8. Krasnikov, S. A., Sergeeva, N. N., Sergeeva, Y. N., Senge, M. O. & Cafolla, A. A. Self-assembled rows of Ni porphyrin dimers on the Ag(111) surface. *Phys. Chem. Chem. Phys.* **12,** 6666-6671 (2010).
9. Masoomi, M.Y., Morsali, A., Dhakshinamoorthy, A. & Garcia, H. Mixed-metal MOFs: unique opportunities in metal-organic framework (MOF) functionality and design. *Angew. Chem. Int. Ed.* **58**, 15188-15205 (2019).
10. Koczkur, K.M., Mourdikoudis, S., Polavarapu, L. & Skrabalak, S.E. Polyvinylpyrrolidone (PVP) in nanoparticle synthesis. *Dalt. Tran.* **44**, 17883-17905 (2015).
