# Supplementary material for: Phase-enabled metal-organic framework homojunction for highly selective CO2 photoreduction
Source: Nat Commun. 2021 Feb 23;12:1231. doi: 10.1038/s41467-021-21401-2 (PMC7902628; doi:10.1038/s41467-021-21401-2)
Supplement: Supplementary file 4 — Description of Additional Supplementary Files [file 41467_2021_21401_MOESM4_ESM.docx]

Description of Additional Supplementary Files

File Name: Supplementary Movie 1

Description: The 3D tomography analysis of Co-MOF-3 nanstack performed using Talos F200X G2. The specimens for 3D tomography observation were cylinders with 0.7 mm in diameter and 5 mm in height. The images of projection were captured at an interval of 0.11° over a total range of 180°.

File Name: Supplementary Movie 2

Description: Super-resolution multi-photon confocal image of Co-MOF-3 was observed in TCS SP8 STED 3X (Leica) with the excitation wavelength of 405 nm, excited from the Diode laser with a power of 50 mW. The emission was collected using a 412-472 nm bandpass filter or a 559-682 nm bandpass filter.
